# Design of CMOS-memristor hybrid synapse and its application for noise-tolerant memristive spiking neural network

**DOI:** 10.3389/fnins.2025.1516971

**Published:** 2025-03-05

**Authors:** Jae Gwang Lim, Sang Min Lee, Sung-jae Park, Joon Young Kwak, Yeonjoo Jeong, Jaewook Kim, Suyoun Lee, Jongkil Park, Gyu Weon Hwang, Kyeong-Seok Lee, Seongsik Park, Byeong-Kwon Ju, Hyun Jae Jang, Jong Keuk Park, Inho Kim

**Affiliations:** ^1^Center for Semiconductor Technology, Korea Institute of Science and Technology, Seoul, Republic of Korea; ^2^School of Electrical Engineering, Korea University, Seoul, Republic of Korea; ^3^Department of Micro/Nano Systems, Korea University, Seoul, Republic of Korea; ^4^Division of Electronic and Semiconductor Engineering, Ewha Womans University, Seoul, Republic of Korea

**Keywords:** neuromorphic hardware, CMOS memristor hybrid synapse, spiking neural network, SPICE simulation, surrogate gradient learning, memristor, artificial synapse

## Abstract

In view of the growing volume of data, there is a notable research focus on hardware that offers high computational performance with low power consumption. Notably, neuromorphic computing, particularly when utilizing CMOS-based hardware, has demonstrated promising research outcomes. Furthermore, there is an increasing emphasis on the utilization of emerging synapse devices, such as non-volatile memory (NVM), with the objective of achieving enhanced energy and area efficiency. In this context, we designed a hardware system that employs memristors, a type of emerging synapse, for a 1T1R synapse. The operational characteristics of a memristor are dependent upon its configuration with the transistor, specifically whether it is located at the source (MOS) or the drain (MOD) of the transistor. Despite its importance, the determination of the 1T1R configuration based on the operating voltage of the memristor remains insufficiently explored in existing studies. To enable seamless array expansion, it is crucial to ensure that the unit cells are properly designed to operate reliably from the initial stages. Therefore, this relationship was investigated in detail, and corresponding design rules were proposed. SPICE model based on fabricated memristors and transistors was utilized. Using this model, the optimal transistor selection was determined and subsequently validated through simulation. To demonstrate the learning capabilities of neuromorphic computing, an SNN inference accelerator was implemented. This implementation utilized a 1T1R array constructed based on the validated 1T1R model developed during the process. The accuracy was evaluated using a reduced MNIST dataset. The results verified that the neural network operations inspired by brain functionality were successfully implemented in hardware with high precision and no errors. Additionally, traditional ADC and DAC, commonly used in DNN research, were replaced with DPI and LIF neurons, resulting in a more compact design. The design was further stabilized by leveraging the low-pass filter effect of the DPI circuit, which effectively mitigated noise.

## 1 Introduction

The exponential growth of data requires the development of efficient hardware systems that consume minimal power while operating at high processing speeds. The von Neumann bottleneck, which is characterized by the separation of processing units and memory, results in a considerable increase in power consumption due to the constant transfer of data between these two components. To address this limitation, a plethora of research has been conducted into and developments have been made in technologies such as ASICs and processing-in-memory (PIM) with the aim of enhancing operations within the von Neumann architecture. Nevertheless, in order to resolve these issues in a fundamental manner, there is an increasing necessity for research into neuromorphic computing, which represents a paradigm shift from the traditional von Neumann architecture (Kemp, 2024).

The deployment of these novel computational architectures presents a number of challenges in relation to throughput, latency and power budget when applied to existing hardware. It is therefore necessary to design specific hardware. The majority of research in this field is based on CMOS technology and can be broadly categorized into two main areas: studies focusing on artificial neural network (ANN) and studies focusing on spiking neural network (SNN). In research based on ANN, the technique of gradient descent is typically employed to adjust the loss, with backpropagation being the primary method for training. Hardware accelerators, specifically neural processing units (NPUs), are developed based on artificial neural networks (ANNs). Functioning between the CPU and memory, NPUs perform parallel processing and large-scale data handling, enabling the rapid processing of bottleneck data and significantly enhancing overall system performance. The development of these accelerator units has reached a point where they are not only utilized in commercial smartphones but also incorporated into laptops (Tan and Cao, 2023; Feng et al., 2024).

In contrast, research based on spiking neural networks (SNN) is primarily concerned with the development of processors that emulate the functionality of the human brain through processing of spatiotemporal spike patterns. Notable advancements have been documented, including the introduction of Intel’s Loihi chip and IBM’s TrueNorth chip. Both chips are designed to include over 1,000,000 neurons and more than 120,000,000 synapses per chip, representing an attempt to replace traditional computing architectures on a fundamental level (Vogginger et al., 2024).

Consequently, there have been continuous efforts to advance and implement neuromorphic computing leveraging CMOS technology. This technology involves the utilization of CMOS-based neuron circuits and synapses, typically implemented using SRAM or DRAM as the foundational synapse elements. However, in terms of area efficiency (GOPS/mm^2^) and energy efficiency (GOPS/W), CMOS based neuromorphic technology generally offers a performance improvement of about one order of magnitude (approximately 10 times) compared to systems driven by GPUs based on conventional von Neumann architectures. The mean values reported in the literature indicate an area efficiency of approximately 300 GOPS/mm^2^ and an energy efficiency of around 400 GOPS/W for the CMOS based neuromorphic systems (Zhang et al., 2020).

Reports indicate that the human brain contains over 10^13^ synapses in the neocortex (Tang et al., 2001). The synapse activity is estimated to occur between 10^13^ and 10^16^ times per second (Merkle, 2007). When this activity is divided by the brain’s power consumption of approximately 25 W, the result is an energy efficiency of around 400,000 GOPS/W (based on 10^15^ operations per second). Further research is required to enhance the area efficiency and achieve power efficiency at the level of the human brain, as well as to reduce volume through stacking.

The current CMOS synapse-based approach to neuromorphic computing is characterized by high power consumption compared to emerging synapse-based neuromorphic computing. Additionally, it requires significant additional circuitry (e.g., ADCs, DACs), and most synapse devices are implemented using SRAM-based designs, which require at least six transistors, leading to limitations in terms of area (Vogginger et al., 2024; Zhang et al., 2020). To overcome these limitations, studies exploring the use of emerging devices for both neurons and synapses have also been reported. A widely adopted approach involves replacing synapses, which account for a significant portion of area and power consumption, with emerging synapse devices, while neurons are commonly implemented using simplified CMOS, several studies on neuromorphic systems based on emerging synaptic devices have demonstrated area efficiencies exceeding 4,000 GOPS/mm^2^ and power efficiencies surpassing 3,000 GOPS/W (Zhang et al., 2020; Mochida et al., 2018; Xue et al., 2019).

Memristors can be classified into several categories, including phase change memory (PCM) (Fong et al., 2017; Burr et al., 2010), magnetic random-access memory (MRAM) (Burr et al., 2010; Tehrani, 2006), ferroelectric random-access memory (FeRAM) (Chen et al., 2020), and resistive random-access memory (RRAM) (of which there are several subcategories, including interface-type RRAM, VCM, and ECM) (Chen, 2020; Ryu et al., 2020). RRAM is distinguished by its stable operation, on/off ratio, speed, and high compatibility with complementary metal-oxide semiconductor (CMOS) technology (Raghavan, 2014; Kim et al., 2021). RRAM offers a number of advantages over traditional DRAM or SRAM, including low power consumption, high operational speed, the ability to store multiple bits of data, and the elimination of the need for refresh, which allows for the construction of large-scale matrices (Dogan, 2013; Perez and De Rose, 2015). Memristors are typically organized in crossbar arrays, wherein each memristor represents a weight value in the matrix. However, crossbar arrays are susceptible to sneak path currents due to Kirchhoff’s law, which has the potential to compromise the accuracy of the network. In order to mitigate the impact of sneak path currents, it is common practice to employ 1T1R structures incorporating transistors (Youssef et al., 2021; Pan et al., 2024).

Memristor-based artificial neural networks (ANN) have been widely documented as hardware accelerators for the recognition and inference of MNIST patterns (Mochida et al., 2018; Xue et al., 2019; Adam et al., 2016; Prezioso et al., 2015). Extensive validation by numerous researchers has also reported the fabrication and validation of memristor chips that are capable of being applied to real-world tasks, including speech recognition, image classification, and motion control (Zhang et al., 2023; Ambrogio et al., 2023). In contrast, memristor-based spiking neural networks (SNN) concentrate on the implementation of innovative neuron structures with the objective of further reducing system power consumption, with the ultimate goal of developing highly efficient and applicable hardware. The application of research on memristor-based SNN chips has been constrained, with the majority of efforts only achieving MNIST inference (Valentian et al., 2019). This highlights the necessity for further investigation into the circuitry architecture and algorithms associated with the relevant hardware (Bouvier et al., 2019).

A significant number of studies employ transistors for the purpose of suppressing sneak paths and acting as selectors. It is therefore essential to exercise caution when selecting transistors for use with memristors, considering the memristor’s operating voltage, resistance characteristics, and the transistor’s current characteristics and on-resistance. The operational characteristics of the memristor are dependent upon whether it is attached to the transistor on the source side (memristor-on-source, MOS) or the drain side (memristor-on-drain, MOD). This must be considered when designing the circuit.

The 2T2R structure consists of two supply voltage lines (each connected to an electrode of the memristor), two gate lines, and a shared source terminal. In this configuration, weights can be implemented with greater flexibility, allowing for the representation of both positive and negative weights. Specifically, one memristor in the 2T2R structure is designated to represent positive weights, while the other represents negative weights. As a result, during weight evaluation, the combined weight is obtained by summing the values of both memristors. In typical implementations, the currents corresponding to positive and negative weights are processed through differential amplifiers or similar circuits, leading to power consumption from both currents. However, the 2T2R structure leverages the opposing directions of the net current flow resulting from the combined weights, allowing the net current to flow directly. This characteristic provides a power-saving advantage over the 1T1R structure, particularly in large-scale neural network implementations (Zhang et al., 2023). Nevertheless, modifying and operating the weights of individual devices in the 2T2R structure requires more complex algorithms. As a result, state-of-the-art research often adopts a hybrid approach, utilizing 2T2R structures for large-scale networks or computationally simple operations and 1T1R structures for regions requiring precise operations. Such hybrid implementations have been reported in recent studies (Zhang et al., 2023).

Implementing neural network arrays with memristors involves numerous considerations, and research focused on the design of transistor-memristor interactions is essential to address these challenges effectively. The operation of a well-designed transistor-memristor array is influenced by several factors, including the accuracy of memristor conductance mapping, which significantly affects the final results as tuning error. In addition to programming errors, intrinsic noise is a major factor that reduces accuracy in neural networks (Zeng et al., 2023; Huang et al., 2023; Park et al., 2021). It is therefore imperative that circuit design techniques which serve to minimize the influence of these factors are employed. As the initial step in optimizing the design of a complete transistor-memristor synapse, it is essential to consider the operating voltage levels of individual memristors and transistors. Consequently, the objective was set to design CMOS devices, known for their higher technological maturity, to align with the operational requirements of memristors. To achieve this, a methodology was proposed to optimize the 1T-1R configuration by designing transistors with variable W/L ratios, thereby enabling the adaptation of transistor characteristics to meet the specific needs of the memristor-based system. An additional consideration involves determining the optimal orientation for attaching the memristor to the transistor. This methodology was utilized to examine the differences and impacts between MOS and MOD configurations, providing insights into the most effective design approach. To further investigate these effects, a compact model was developed with characteristics identical to those of the fabricated memristor. This was used in conjunction with a design of SNN hardware, including a 1T1R array, a differential-pair-integrator (DPI) synapse circuit, and a leaky-integrate-fire (LIF) neuron, to implement an inference accelerator in a circuit level. SNN simulations by SPICE were conducted on the designed memristive neural networks of a small scale (8 × 8), considering both tuning errors and intrinsic noise. The findings of this study thus lead to the proposal of an optimal design for noise-tolerant memristive-SNN hardware, and to the demonstration of the advantages of using SNN for high-efficiency computing in comparison to ANN.

## 2 Methods

### 2.1 Fabrication of memristive devices

The memristor single element was fabricated with a cross bar array structure and composed of Cu:Te/TaO_x_/IGZO/Pt. The substrate of the element was a thermally oxidized c-Si wafer (300 μm), and was ultrasonically cleaned in acetone, ethanol, and deionized water for 10 min each before fabrication. Each layer was patterned using lithography, and an image reversible photoresist was used. After deposition, the residual photoresist was etched using the lift-off method. The bottom electrode had a line width of 32 μm and was deposited using an electron beam evaporator. The first deposition involved inserting 5 nm of Ti for adhesion between the substrate (SiO_2_) and the bottom electrode (Pt). Afterwards, 25 nm of Pt was deposited without vacuum break. Then, a sputter was used to deposit a buffer layer. The composition of the IGZO target is In_2_O_3_:Ga_2_O_3_:ZnO =1:1:1, and it was deposited under an oxygen partial pressure of 0.1% by controlling the mixed gas of Ar and O_2_ (40 sccm). The process pressure is 4 mTorr and the power is 50 W. The pattern used was a square model of 100 μm x 100 μm, and the thickness was 100 nm. The switching layer was deposited using a TaO_x_ ceramic target and a sputter was used. It was deposited at a working pressure of 3 mTorr in an Ar (40 sccm) atmosphere without oxygen gas. The pattern used was a square model of 300 μm x 300 μm, and the thickness was 5 nm. Finally, the top electrode, Cu:Te composite layer, was deposited using an e-beam evaporator and a line width of 32 μm pattern was used. A total of 30 nm of the top electrode was deposited through alternative deposition of Cu 3 nm and Te 2 nm, and 10 nm of Au was deposited to prevent oxidation. All layers using the E-beam evaporator were maintained at a base pressure of 2 × 10^−7^ torr and an acceleration voltage of 7.2 kV. In addition, layers using the sputter were maintained at a base pressure of less than 5 × 10^−7^ torr.

The current–voltage characteristics of the devices were assessed using a Keithley 4200A-SCS parameter analyzer with source measurement unit (SMU). For the single memristor devices, voltage was applied to the Au/Cu:Te top electrode and the bottom electrode Pt was grounded. The initial electro-forming process of the device was 2 V, sweep sequence was proceeded a fully LRS from 0 V to 2 V, multi-level state was achieved by varying the reset stop voltage. Compliance current is not set due to the device’s self-limiting behavior. The reset stop voltage gradually increased and swept from −0.5 V to −2 V at −10 mV intervals. At this time, after completing the reset stop voltage sweep operation, a read operation (@0.05 V) was performed for 5 s to check the conductance difference from the previous level. If is less than 0.2 μS, the reset stop voltage has been increased further. All reset sweep operations were carried out without set operation (2 V). Finally, to check the retention behavior of each level, it was read with a reading voltage of 0.05 V for 100 s. The reading interval is 0.01/s.

### 2.2 Fabrication and operation of 1T1R synapse

In a typical 1T1R structure, NMOS transistors are typically preferred due to their use of electron carriers, which provide a mobility that is 2–3 times higher than that of PMOS transistors (Streetman and Banerjee, 2000). Furthermore, the use of a p-type substrate for NMOS transistors eliminates the necessity for an additional n-well process step (Raj and Latha, 2008), which is advantageous from a fabrication standpoint. As a result, NMOS transistors were selected, and the technology from the ETRI 500 nm commercial Si foundry in South Korea was utilized. A total of eight photomask layers were fabricated, and transistors with varying W/L ratios, comprising six different types, were produced (Streetman and Banerjee, 2000).

**Figure 1:**
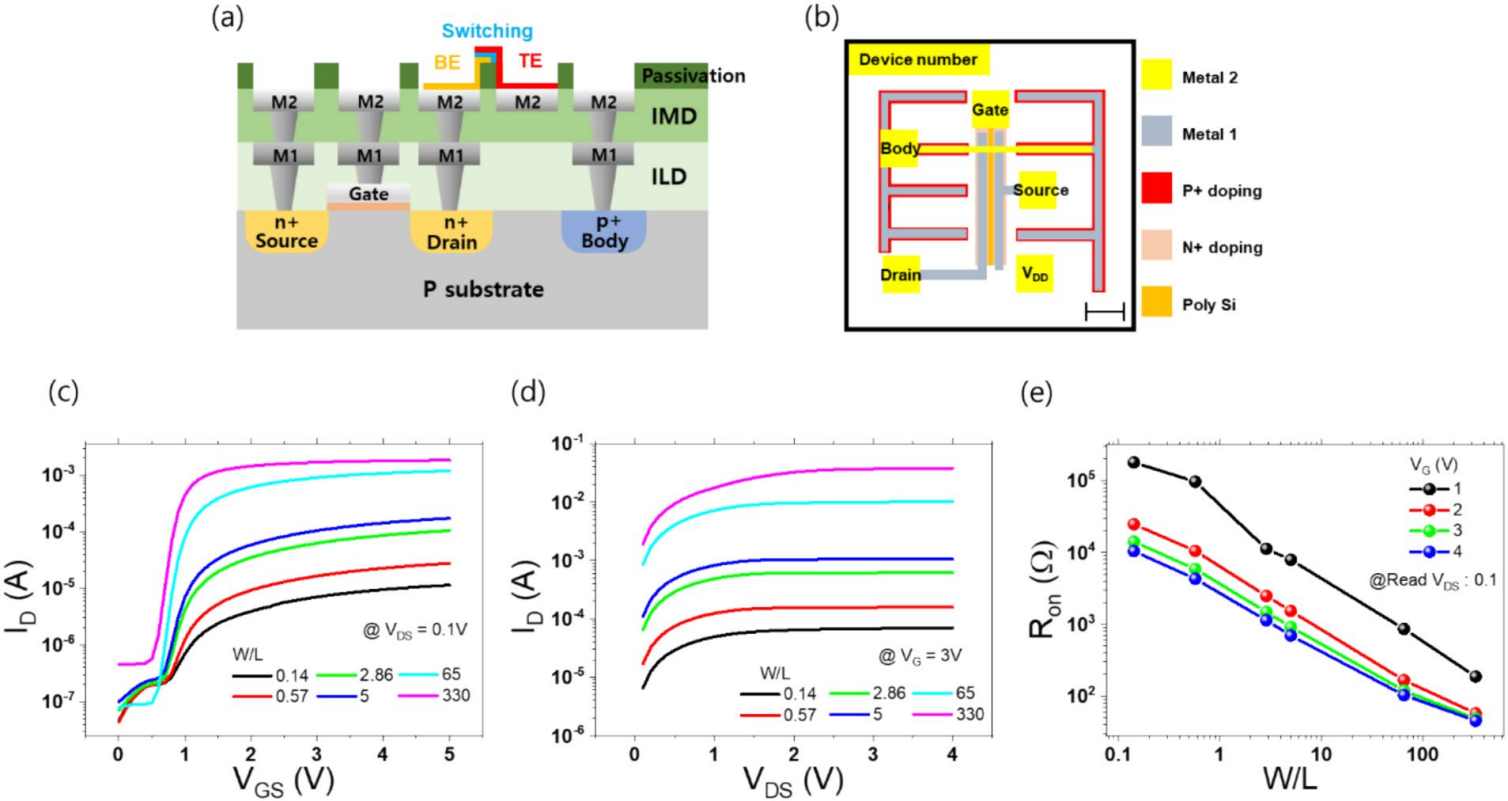
Panel **(A)** presents the cross-section of the transistor designed through the ETRI process. Panel **(B)** illustrates the layout of the transistor, showing the five pads corresponding to Gate, Body, Source, Drain, and V_DD_. The detailed layers, including Metal 2, Metal 1, P+ doping, N+ doping, and Poly-Si, are depicted, with VIA and passivation layers omitted. The length of the scale bar in the bottom right is 110 μm. Panel **(C)** displays the transfer curves measured by applying a *V*_DS_ of 0.1 V and varying the gate voltage for six transistors with different W/L ratios. Panel **(D)** shows the output curves for the six transistors under a gate voltage of 3 V, plotting the drain current against varying *V*_DS_. Panel **(E)** presents the on-resistance of the transistor as a function of W/L and gate voltage, measured at a *V*_DS_ of 0.1 V.

Each transistor was designed with four pads, which were used for the connection of the transistor to the external circuitry. The source, gate, drain, and body are the four main components. Furthermore, a V_DD_ pad for the memristor’s electrode was incorporated to facilitate integration with memristors utilizing BEOL processing at the KIST fab. Consequently, each unit transistor was equipped with a total of five pads. The cross-section schematic and circuit layout of the 1T1R structures are shown in Figures 1A,B, respectively. As depicted in Figure 1A, a *V*_DD_ pad utilizing only metal 2 layer was designed to monolithically place a memristor between the drain pad and the *V*_DD_ pad. The finalized TEM and OM images are presented in Supplementary Figure S1.

Subsequent electrical measurements of the completed transistors and 1T1R structures were conducted using a Keithley 4,200 SMU. In conducting the transistor measurements, positive voltages were applied to the gate and drain pads, while the source and body pads were grounded. Transfer and output curves were obtained by varying the voltage. The transfer curve was obtained by fixing the drain voltage at 0.1 V and varying the gate voltage from 0 to 4 V. The output curve was obtained by fixing the gate voltage at 3 V and varying the drain voltage from 0 to 4 V. The resulting IV curves are presented in Figures 1C,D. To provide a comprehensive overview of the impact of varying W/L ratios and gate voltages, the on-resistance graph for each transistor condition is presented in Figure 1E.

Following verification, the transistors were employed in the fabrication of 1T1R structures via BEOL processing. In the fabrication of 1T1R structures, the application of positive voltage was contingent upon the attachment direction of the memristor. This voltage was applied to either the top electrode (TE), the bottom electrode (BE), or the gate pad, while the source and body pads were grounded.

**Figure 2:**
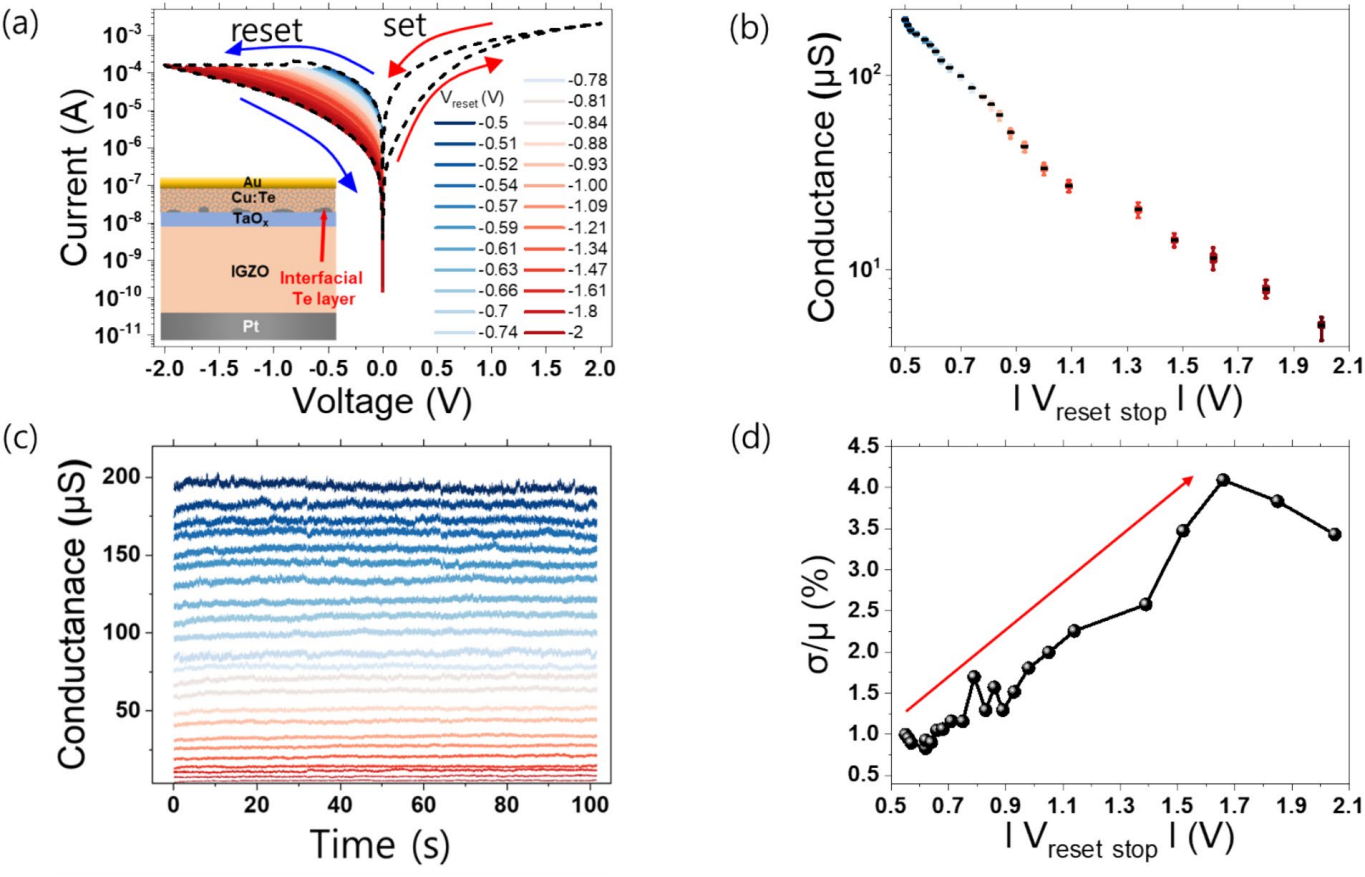
**(A)** Schematic illustration of single memristor device, and I–V characteristic result for each reset voltage sweep range. The dash lines are fully set 2 V or fully reset −2 V sweep curves, respectively. The blue to red lines are sweep curves formed by increasing the reset stop range after set. **(B)** Conductance level distribution at read voltage 0.05 V for each reset voltage sweep range. **(C)** Retention and noise behavior at 0.05 V read voltage in 100 s for each conductance level. The colors of each conductance level correspond to the *V*_reset stop_ colors in **(A)**. **(D)** Relative standard deviation of the **(C)** result.

In order to conduct closed-loop conductance tuning of the 1T1R structures, feedback control using LabVIEW was necessary, and measurements were taken using NI instrumentation. Two NI 4139 (20 W) SMU models were utilized: one for the application of DC voltage to the gate of the 1T1R, and the other for the application of voltage to the TE, with the opposite electrode connected to ground. Utilizing the measured current, incremental step pulse programming (ISPP) was employed to augment the voltage by delta V increments, applying both positive and negative voltages until the current reached the target value within a specified error margin (Figures 2D,5C).

### 2.3 Memristor compact model and circuit-level SPICE simulation

The final hardware-based circuit was simulated using the LTSpice (x64) version 17.1.8 software. In light of the necessity of employing external PWL files as input signals and reflecting the weights derived from Pytorch simulations into SPICE, it became evident that substantial modifications to the LTSpice netlist files were required. Consequently, the simulations were conducted exclusively within the Python 3.11.5 environment, utilizing the PyLTSpice package. The PyLTSpice package, developed by electronic engineer Nuno Brum, employs the spicelib library within the Python programming language to facilitate the editing of LTSpice netlists, the identification of specific command lines, the modification of simulation conditions, and the examination of simulation results. The latest version of the package, 3.0, has been released and its command functionalities are documented on GitHub and the developer’s personal website (Brum, n.d.).

The equations, structure, and I-V curve of the memristor compact model are detailed in Supplementary Figure S2. Furthermore, fluctuations in the values of *I*_0_ and *R*_s_ were incorporated into the model using the Gaussian function in LTSpice. The IV curves for 10 cycles, with variations of 1, 3, 5, 10, 30, and 50%, are presented in Supplementary Figure S3.

### 2.4 Pytorch SNN simulation

A network simulation was conducted using the Python programming language with the PyTorch framework (Paszke et al., 2019). The pattern recognition task employed the reduced MNIST dataset (Alpaydin, 1998), comprising handwritten digit data from 43 individuals, spanning the range of digits from 0 to 9. The dataset was then converted into grayscale images with a resolution of 32 × 32 pixels and subsequently downsampled to a resolution of 8 × 8 pixels. The latest version of the package, 3.0, has been released 8 pixels by aggregating 4 × 4 pixel blocks into single pixels. The pixel intensity was represented in 16 levels, ranging from 0 to 15. The total number of images was 5,620, with 80% (4,496 images) allocated for the training dataset and 20% (1,124 images) designated for the test dataset.

The neural network for the reduced MNIST dataset had 64 inputs, corresponding to the 8 × 8 pixel images, and 10 outputs, which were used to classify the handwritten digits from 0 to 9. In the ANN simulation, the pixel intensity values were input into a single-layer perceptron structure comprising 64 input neurons and 10 output neurons. Subsequently, the output signals were subjected to a softmax function (Paszke et al., 2019), thereby determining the probability distribution for each class. Based on the PyTorch code described, simulations were conducted. The loss was calculated by comparing the predicted labels with the actual labels, and the weights were updated using the Adam optimizer through backpropagation over 30 epochs. For the same dataset, the DNN achieved an accuracy of 95%, while the surrogate SNN attained an accuracy of 90%.

There are numerous models for simulations of spiking neural networks (SNN). These can be broadly categorized into three types. The first involves transferring weights from an ANN after training and converting input and output signals to spike signals for inference (Han and Roy, 2020). The second method entails the utilization of teaching signals or backpropagation for the purpose of training the SNN (Wu et al., 2019; Shen et al., 2022; Neftci et al., 2019). The final approach makes use of biologically plausible methods, such as spike-timing-dependent plasticity (STDP), for the purpose of training (Diehl and Cook, 2015; Dong et al., 2023). These methods are investigated with an increasing focus on their biological plausibility. Among the SNN methods that employ backpropagation, surrogate gradient learning represents a notable approach. As spike signals are discrete and non-differentiable, the computation of errors and backpropagation are not feasible, which in turn prevents weight updates and hinders learning. To address this challenge, Emre O. Neftci’s paper proposed (Neftci et al., 2019) a method utilizing LIF neurons in hardware. In this approach, synapse currents facilitate the conversion of signals over time, while membrane potentials undergo stepwise changes. While the forward spike signals are transmitted in the usual manner, the backpropagated spikes are converted to surrogate gradient signals in order to compute the loss and update the weights. Therefore, in our study, surrogate learning was employed to implement learning based on the SNN.

The neuron, a key component, was modeled using a simplified RC circuit, where the membrane potential is represented by a capacitor and the leaky current by a resistor. To simplify the computation in PyTorch, we encapsulated the effects of membrane resistance and capacitance into a single parameter, avoiding the need to explicitly model each component. This approach allowed for efficient simulation of neuronal dynamics. Additionally, to accurately capture the biological characteristics of synaptic responses—specifically, the rising and falling dynamics of synaptic current as observed in biological synapses—we employed a double exponential synapse model on the LIF framework. The input signals were generated using the PyTorch spikegen function (Paszke et al., 2019), with the firing times determined by pixel intensity. The network was trained for 30 epochs with a batch size of 5, resulting in an accuracy of 90%. All simulations were conducted with positive weights only, with values normalized between 0 and 1.

## 3 Results and discussion

### 3.1 Multi-level memristor device

Figure 2A shows a cross-sectional view of a single unit memristor device illustration intended for use in the memristive neural network and its resistive switching. When a positive bias is applied to the top electrode, Cu migrates toward the bottom electrode to form a filament. Conversely, when a negative bias is applied to the top electrode, the Cu filament dissolution occurs and migrates back toward the top electrode. The existence of the Te interfacial layer restricts the Cu migration path, enabling stable switching behavior (Goux et al., 2011). In addition, the alloy of Cu and Te efficiently suppresses excessive Cu migration, improving endurance (Tseng et al., 2018). The gradual resistance change characteristic is essential for implementing multi-conductance levels. In a multi-level state, each resistance state must be precisely defined. Gradual switching characteristics allow fine modulation of resistance through gradual resistance changes, rather than abrupt resistive switching characteristics, making it easy to set a specific desired resistance state and implementing various intermediate resistance states. Therefore, IGZO based Cu:Te device exhibits gradual resistance change behavior because it exhibits multi-weak filament characteristics rather than strong single filament. The number of pulses required to transition from the initial conductivity state to the minimum and maximum conductivity. During the electro-forming process of the device, the presence of IGZO, a buffer layer, creates a heat confinement effect so multi filaments are induced within the switching layer (Gao et al., 2017). At this time, multiple filaments are formed sequentially, resulting in gradual switching behavior. If only a partial reset is achieved by reducing the reset voltage rather than fully resetting (−2 V), the conductance level can be modulated by controlling the number of multi filaments.

In order to show analog multi-level characteristics in IGZO-based Cu:Te devices, applying negative voltage (*V*_reset_) sweep up to specified conductance values and presented in Figures 2A,B, respectively. Due to the gradual reset behavior of the device, conductance can be modulated at various negative voltages. The multi-level formation process is as follows. First, a −0.5 V reset was performed to form the initial level (*G*_0_), and then a reset operation was performed by adding δV to the current reset voltage when forming the next level (*G*_1_). At this time, the initial δV value was −1 mV, and to clearly distinguish between levels considering the C2C and D2D variations when forming the next level, a 3-s read operation was performed. If the conductance difference between the current level (*G*_n_) and the next level (*G*_n + 1_) was less than 0.2 μS (ΔG = *G*_n, min_ – *G*_n + 1, max_ < 0.2 μS) during the 3-s read operation, the reset process was performed again by increasing −1 mV from the current δV. This level formation process was performed until the fully reset voltage of 2 V was reached without a set process. Figure 2C show the multi-level behavior obtained in Figure 2A through a read operation (0.05 V) for 100 s. As shown in Figure 2C, 23 multi-level states were formed in 5.7–200 μS due to various reset stop sweep operations (−0.5 V to −2 V). Each conductance level obtained through various reset stop sweep operations (−0.5 V to −2 V) was maintained constant without degradation, and the interval between levels was modulated to be at least 0.2 μS. Figure 2D shows the relative standard deviation (RSD) of the multi-levels obtained through the read operation. As the reset stop voltage increases and the conductance level decreases, the RSD tends to increase. This means that random telegraph noise (RTN) intervention is different for each conductance level and the number of multi-level states is modulated by IGZO-based Cu:Te devices (Veksler et al., 2013). Therefore, if the conductance level is low, the number of conductive filaments in the switching layer and the number of Cu atoms in the constriction area of each filament will decrease. Therefore, the probability of electrons being trapped/de-trapped by the charged instable filament around the conductive filament will increase (Belmonte et al., 2014; Rao et al., 2023).

### 3.2 Design of 1T1R synapse and its operation for multilevel conductance tuning

**Figure 3:**
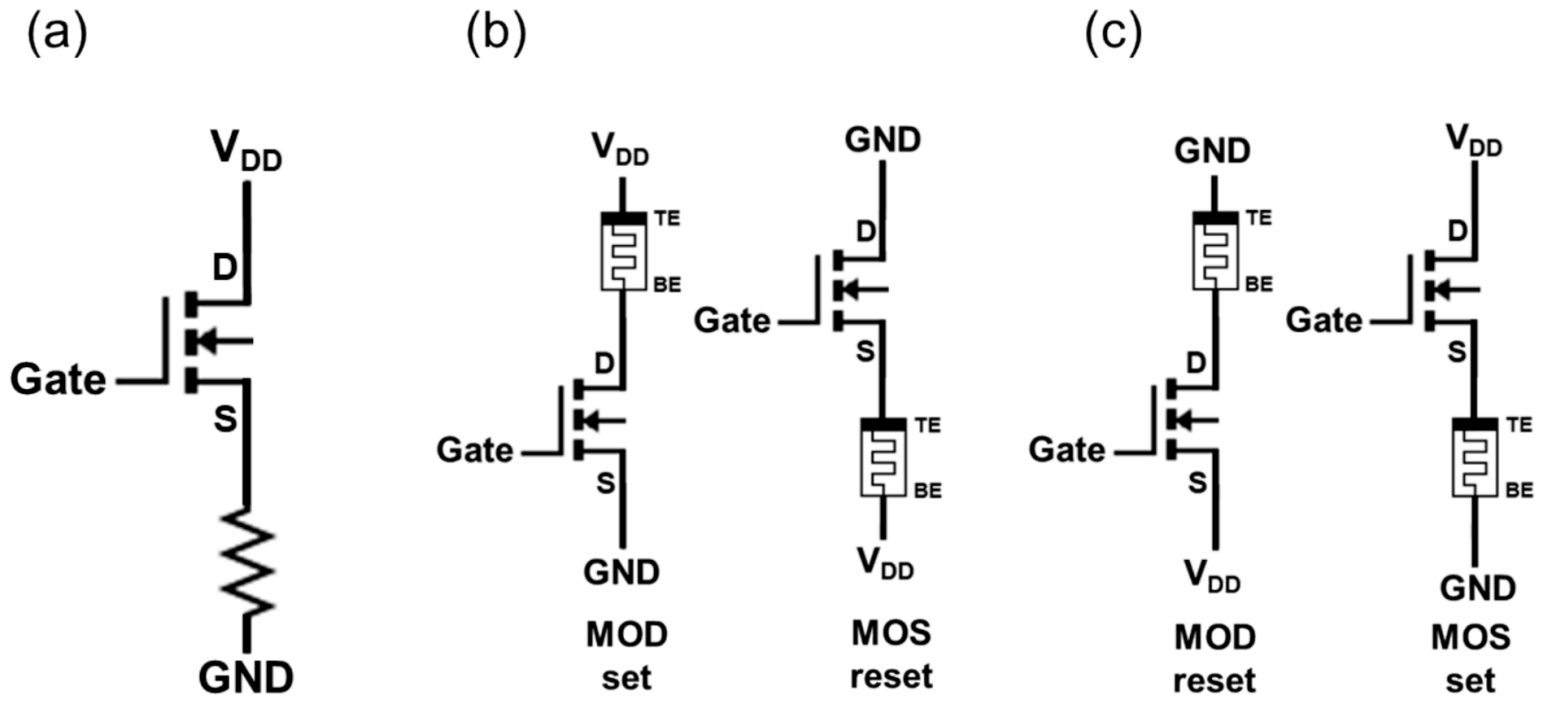
Panel **(A)** presents the schematic of a memristor fabricated through the BEOL process after transistor design. Panel **(B)** shows the 1T1R schematic in the MOD set and MOS reset conditions. Panel **(C)** illustrates the 1T1R schematic in the MOD reset and MOS set conditions.

The attachment of a memristor to a transistor through BEOL processing results in the formation of the structures shown in Figure 3A, wherein the resistor is situated either at the transistor’s drain or source (Ghenzi et al., 2018; Maheshwari et al., 2021). These configurations are designated as “memristor on drain” (MOD) and “memristor on source” (MOS), respectively, as illustrated in Figures 3B,C.

The transistor, acting as a selector, ideally must transmit the full supply voltage (*V*_DD_) to the memristor. Additionally, it must possess an off-resistance greater than that of the memristor to effectively suppress sneak path currents. While a transistor is, in theory, capable of functioning as a switch without resistance, a number of practical considerations must be taken into account. These include the operating regions of the transistor, which may be either a triode or in saturation. One such factor is the attachment orientation of the memristor. In the case of a MOD configuration with a positive supply voltage, where the bottom electrode of the memristor is attached to the NMOS’s drain terminal, the supply voltage V_DD_ is divided into both of the transistor and the memristor depending on their resistances. From the memristor’s point of view, this results in a loss of the supply voltage due to the voltage drop across the transistor. Accordingly, a transistor with an appropriate on-resistance should be selected based on the current required for memristor operation. The memristor, a variable resistor whose resistance changes with voltage, is represented as a resistive element in the 1T1R structure illustrated in Figure 3A.

The memristor, while often simplified as a fixed resistor, is in fact a bipolar device with two terminals: an anode and a cathode. The 1T1R operation conditions depend on both the memristor attachment configuration and the bias polarity, rather than being a simple transistor-resistor relationship. Specifically, the set behavior of the memristor in the MOD configuration and the reset behavior in the MOS configuration exhibit structural and operational symmetry as illustrated in Figure 3B. Similarly, the reset behavior in the MOD configuration and the set behavior in the MOS configuration also demonstrate identical voltage application methods and structural characteristics, as depicted in Figure 3C. Effective memristor operation requires facilitation of both set and reset operations. In the fabrication of 1T1R devices, achieving a monolithic structure is crucial to eliminate unnecessary auxiliary circuits and simplify the design. Since the orientation of the memristor (MOD or MOS) is predetermined during manufacturing, careful selection of the attachment orientation is essential to avoid operational interference (Liu et al., 2024; Bengel et al., 2023).

Before explaining the differences caused by the attachment direction, it is important to note that in a typical 1T1R configuration, the voltage drop across the memristor typically exceeds the voltage drop over the transistor (V_DS_) when the transistor operates in the triode region. The following discussion is based on this condition. In the MOD configuration, the applied V_DD_ voltage is applied to the memristor with a negligible transfer loss if the gate-to-source (V_GS_) is over the transistor threshold voltage (V_th_). Conversely, in the MOS configuration where the memristor is attached to the source of the transistor, applying a high voltage to the V_DD_ pad and grounding the BE (bottom electrode) of the memristor (MOS set case) results in the transistor’s source voltage (V_S_) equating to the memristor’s top electrode voltage (V_TE_). Consequently, V_GS_ becomes V_G_ − V_memristor_, requiring a higher gate voltage to turn on the transistor. When the gate voltage is V_DD_ (V_G_ = V_DD_), the maximum voltage drops across the memristor are V_DD_ – V_th_.

These relationships differ depending on whether a memristor is in the set or reset state, as summarized in Table 1. If the memristor requires a higher set voltage than a reset voltage, the MOD configuration, which minimizes a voltage transfer loss across the transistor during the set operation, is advantageous. Conversely, if a higher reset voltage than a set voltage is required, the MOS configuration, which minimizes a voltage loss during the reset operation, is preferred. Therefore, research groups designing and fabricating 1T1R arrays must determine whether to use the MOS or MOD configuration in advance. For our Cu:Te-based memristor devices, which exhibit a gradual set characteristic, a set voltage of up to 2 V is required. As demonstrated in Figures 2A,C, analog states were achieved under full set conditions by adjusting the reset stop voltage. Hence, fully setting the memristor is an essential requirement. To meet this condition, we adopted the MOD configuration, which minimizes the voltage transfer loss over the transistor during the set operation. After determining the 1T1R configuration, the operation conditions analysis for the memristor switching was performed based on the W/L ratio and the resistance of the memristor. The detailed analysis results are provided in Supplementary Figure S4.

**Table 1:** The table provides a summary of the maximum voltage that can be applied to the memristor (*V*_memristor_), determined by the 1T1R configuration.

Voltage on memristor (*V*_memristor_)	SET	RESET
Memristor on drain (MOD)	V_DD_	V_DD_ – V_th_
Memristor on source (MOS)	V_DD_ – V_th_	V_DD_

**Figure 4:**
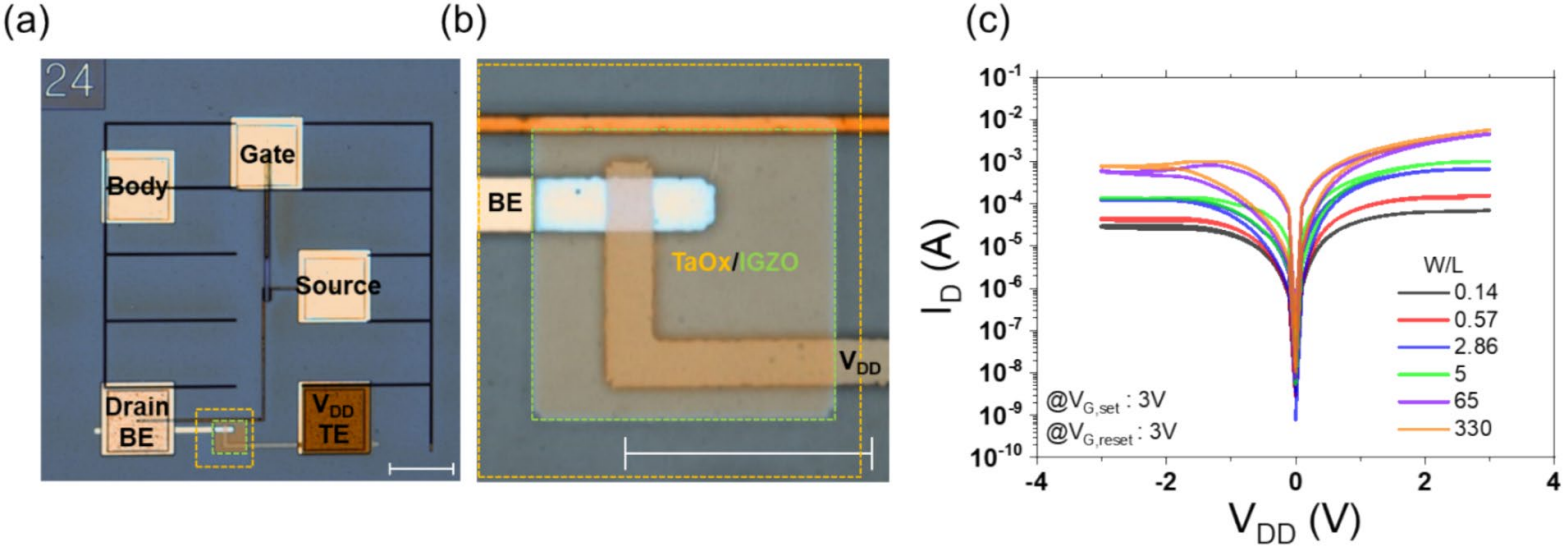
Panel **(A)** shows an optical microscope (OM) image of the monolithic 1T1R device fabricated between the Drain pad and V_DD_ pad through the BEOL process, with a scale bar representing 100 μm. Panel **(B)** presents a magnified OM image of the region where the top electrode (TE) and bottom electrode (BE) of the memristor intersect. The switching layer and buffer layer are marked in orange and green, corresponding to TaOx and IGZO, respectively. The scale bar represents 50 μm. Panel **(C)** displays the *V*_DD_ voltage vs. Drain current IV curve for the monolithic 1T1R device, as previously shown in the OM images, across varying W/L ratios. The gate voltage is fixed at 3 V during the set and reset measurements.

Considering these findings, the MOD configuration was selected as the optimal operating mode, and for ease of set and reset operations, the device was designed with a W/L ratio of at least 65. To confirm the correct operation of the 1T1R device, we constructed it by depositing the BE on the drain pad, followed by the switching layer, and finally the TE pad. The optical microscope (OM) images of the fabricated device are presented in Figures 4A,B. Figure 4A depicts the overall view, while Figure 4B shows a magnified view of the switching layer. The fabricated device was tested by sweeping *V*_TE_ while the gate voltage was set to 3 V for both set and reset operations. As illustrated in Figure 4C, when the W/L ratio is at least 65, the set behavior is comparable to that of the memristor alone, and the reset operation is also performed smoothly, achieving an on/off ratio of approximately 87.5% in comparison to the unit device. The 10% loss can be attributed to the on-resistance (*R*_on_) value of 120 Ω for a width-to-length (W/L) ratio of 65, which results in a 12% voltage drop across the transistor relative to the memristor’s low-resistance state (LRS) of 1 kΩ. It is therefore proposed that a transistor with an on-resistance within 10% of the memristor’s resistance will facilitate optimal operation. In light of the considerable increase in size for a larger transistor model (W/L ratio of 330) and the flexibility of operating the transistor in triode and saturation modes with a W/L ratio of 65, the decision was made to select a transistor with a W/L ratio of 65. The IV results for the memristor on source configuration and the on/off ratio variations with W/L changes are presented in the Supplementary Figure S5 and Supplementary Table S2. Supplementary Figure S6 illustrates the analogous IV curve obtained through the utilization of the compact model for 1T1R measurements, encompassing both MOS and MOD configurations.

**Figure 5:**
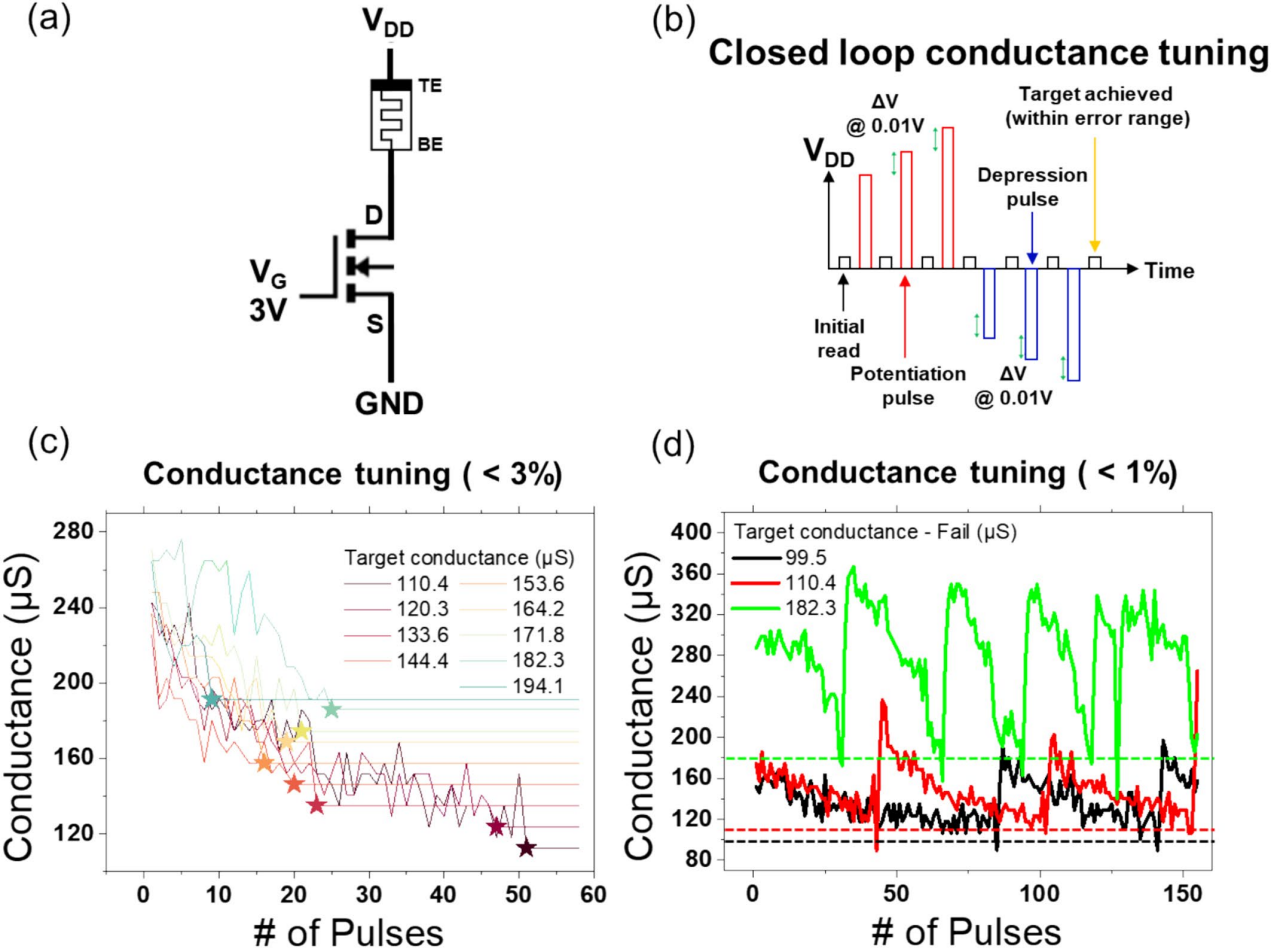
Panel **(A)** shows the 1T1R schematic of the MOD structure for conductance tuning, where the gate voltage is fixed at 3 V, and the *V*_DD_ voltage is adjusted. Panel **(B)** illustrates the voltage profile applied to *V*_DD_ for conductance tuning. A voltage of 0.05 V is used to read the current conductance state, which is then compared with the target value. Based on this comparison, either a potentiation pulse or a depression pulse is applied, and the process is repeated. The amplitude of the potentiation and depression pulses is continuously varied by Δ*V* (0.01 V) until the target conductance is reached. The initial potentiation pulse voltage is 2 V, while the depression pulse voltage is set according to the conditions in Figure 2A based on the target conductance. Panel **(C)** demonstrates that the closed-loop conductance tuning algorithm successfully achieved 9 target conductance values within a 3% error margin using 60 pulses or fewer. Panel **(D)** shows that when the error margin is tightened to 1%, the algorithm fails to achieve the target conductance, even after more than 150 pulse iterations.

The structure of the NMOS and memristor used for conductance tuning is shown in Figure 5A. The gate voltage was fixed at 3 V, the source voltage was grounded, and the top electrode voltage was varied during the process. A closed-loop conductance tuning procedure was conducted using the 1T1R cell in the MOD configuration with a W/L ratio of 65. The fundamental algorithmic process is depicted in Figure 5B. By applying an initial voltage of 2 V to fully set the memristor and then adjusting the initial reset voltage based on the unit memristor’s conductance, the target conductance was successfully identified. With a ΔV of 0.01 V and an error range of 3%, nine conductance tunings were achieved within 55 pulses, as illustrated in Figure 5C. However, when the error range was decreased to 1%, the system was unable to identify the target conductance, exhibiting a repetitive switching between set and reset states, as illustrated in Figure 5D. This outcome suggests that the intrinsic noise of the unit memristor, estimated to be approximately 2%, may have hindered the successful identification of the target value within the specified 1% error range. Furthermore, even if the target value were to be successfully identified, the intrinsic noise would likely introduce instability, necessitating repeated loop executions.

### 3.3 Circuit-level design of a neuron-synapse-neuron unit for spiking neural networks

**Figure 6:**
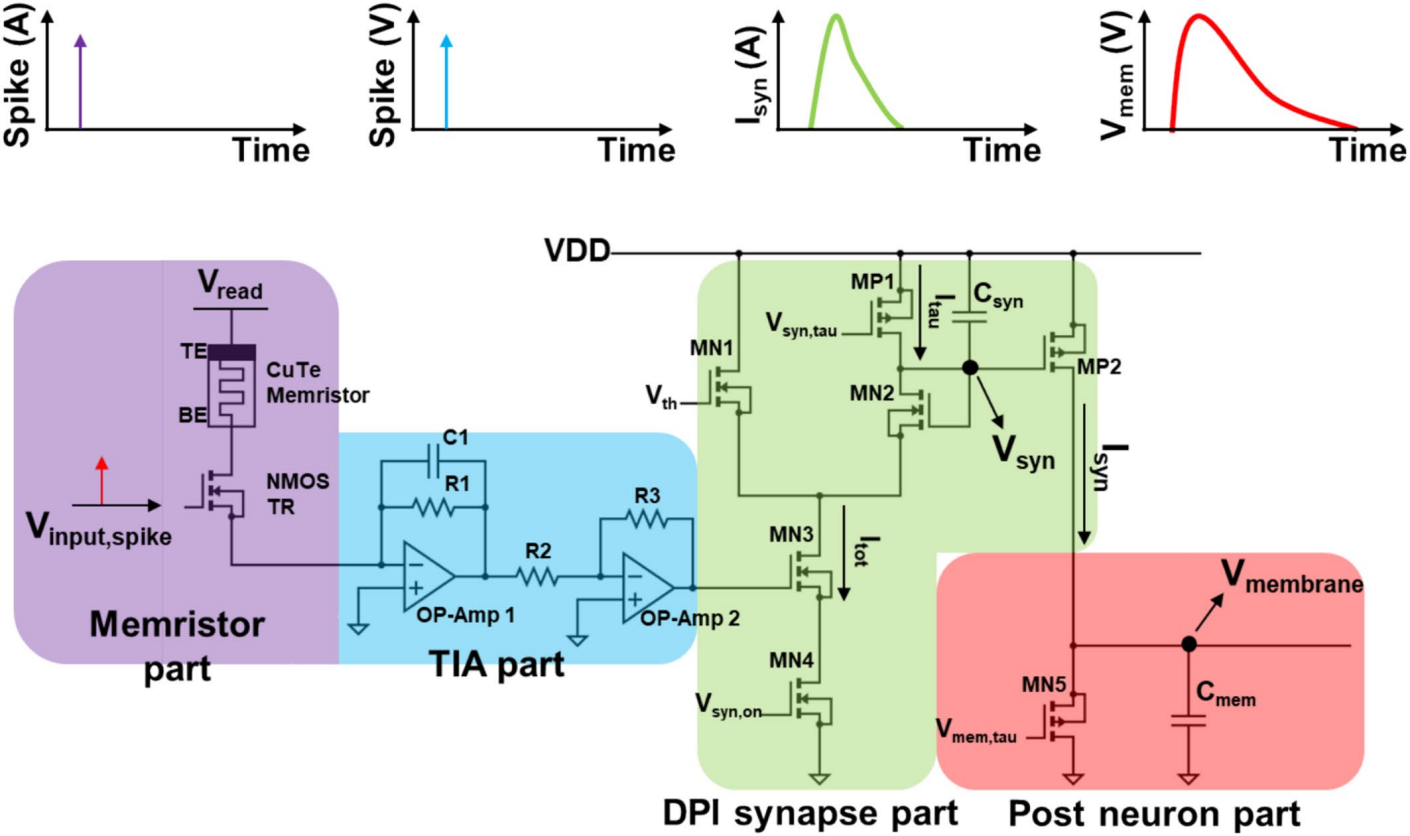
The circuit structure that mimics biological behavior in a Neuron-Synapse-Neuron configuration consists of four parts: the Memristor part, TIA part, DPI synapse part, and Post-neuron part. The Memristor part features a 1T1R structure that converts the input spike (*V*_input, spike_) into a current spike based on the memristor’s weight. In the TIA part, the current spike is converted into a voltage spike signal. The DPI synapse part processes the voltage spike, incorporating the weight, and converts it into synaptic current (*I*_syn_) following the double exponential rule through the charging and discharging of *C*_syn_, resulting in changes in *V*_syn_. Finally, in the Post-neuron part, *I*_syn_ drives the charging and discharging of *C*_mem_, leading to the membrane potential of the neuron, represented as *V*_membrane_.

In accordance with the previously established 1T1R configuration, a verification process was undertaken at the unit level of neuron-synapse-neuron (N-S-N) prior to the execution of the comprehensive network simulation. As illustrated in Figure 6, the single N-S-N network circuit was implemented using a 1T1R synapse, a TIA circuit, a DPI circuit and a LIF neuron circuit. The DPI circuit was introduced to emulate a dynamic synapse current behavior. Biological neurons transmit information through electrical or chemical synapses (Petersen, 2016). Electrical synapses directly connect and allow current flow between two neurons through gap junctions. However, the most common synaptic mechanism is chemical synapses (Pereda, 2014). In chemical synapses, the generated spike signal antidromically propagates to the axon terminal, triggering synaptic vesicle exocytosis and subsequently release neurotransmitters. When the released neurotransmitters cross the synaptic cleft and bind to postsynaptic receptors, postsynaptic ion channels such as AMPA or GABA receptors open. This alters the ionic permeability such as Na^+^, Ca^2+^ or Cl^−^, and subsequently depolarizing or hyperpolarizing the membrane potential of the dendrites forming synapse. Since these steps are highly dynamic due to chemical diffusion and reaction of neurotransmitters, a synaptic response model that evokes postsynaptic current using a unit function input without considering any synaptic current cannot accurately describe the synaptic response in the postsynaptic neuron. Moreover, even when employing a synaptic current model of a single exponential model that only considers the decay phase of postsynaptic current fails to fully capture the rising dynamics synaptic current (Rothman and Silver, 2014). Therefore, in most studies, postsynaptic responses are commonly described using a double exponential, where one exponential for the rising phase and another for the decay phase of the synaptic response (Beniaguev et al., 2021; Jang et al., 2020; Tikidji-Hamburyan et al., 2023). The double exponential synapse dynamic behavior can be emulated in numerical simulation using the following equations of discrete forms:


(1) $$\mathrm{Normalize}\_\mathrm{factor}\left(N_{F}\right)=\frac{\tau_{syn\left(\mathrm{decay}\right)}}{\left(\;\tau_{syn\left(\mathrm{decay}\right)}-\tau_{syn\left(\mathrm{rise}\right)}\;\right)}$$

(2) $$I_{syn\_\mathrm{new}\left(\mathrm{rise}\right)}=\tau_{syn\left(\mathrm{rise}\right)}\cdot I_{syn\left(\mathrm{rise}\right)}+\left(S\left(t\right)\cdot W_{n}\right)\cdot N_{F}$$

(3) $$I_{syn\_\mathrm{new}\left(\mathrm{decay}\right)}=\tau_{syn\left(\mathrm{decay}\right)}\cdot I_{syn\left(\mathrm{decay}\right)}+\left(S\left(t\right)\cdot W_{n}\right)\cdot N_{F}$$

(4) $$I_{syn\_\mathrm{total}}=I_{syn\_\mathrm{new}\left(\mathrm{decay}\right)}-I_{syn\_\mathrm{new}\left(\mathrm{rise}\right)}$$

(5) $$V_{mem\_\mathrm{new}}=\tau_{mem}\cdot V_{mem}+I_{syn\_\mathrm{total}}$$

The application of the double exponential model requires the definition of the normalize_factor in accordance with the specifications set forth in Equation 1. The calculation of the rise and decay synaptic currents is performed by considering *τ*_syn(rise)_ and τ_syn(decay)_ through Equations 2, 3, respectively. Equations 2, 3 are composed of different terms, including 𝜏_syn_, S(t), and *W*_n_ and normalize_factor. S(t) represents the spike generated when the membrane potential exceeds the threshold, indicating the occurrence of a spike under that condition. The *W*_n_ value corresponds to the weight, and it can be observed that a higher weight leads to a larger current flow, even for the same spike, depending on the equation. The total synaptic current, 𝐼_syn_total_, is then obtained using Equation 4, which accounts for the time constants of both the rise and decay phases. As outlined in Equation 5, the membrane potential rises in response to the synaptic current and decays in accordance with the membrane time constant, 𝜏_mem_.

To achieve emulation of the double exponential synapse behavior in the neuron-synapse-neuron (N-S-N) network, adjustments were made to the tau-related gate voltages in the DPI circuit, ensuring that the current variation corresponding to the memristor’s state remained consistent. Upon the application of input spikes to the gate of the NMOS transistor, the current flows in accordance with the conductance of the memristor, with *V*_read_ set to 50 mV. The current is then amplified by the TIA using operational amplifier 1, and the inverted output voltage is applied to the gate of the MN3 transistor in the DPI circuit via operational amplifier 2. In 1989, Mead put forth a circuit that emulates a pulsed current source synapse, whereby synapse current is conducted when pulse signals are applied. This circuit has undergone continuous improvement, with the current form of the DPI proposed by Bartolozzi and Indiveri (2007), fabricated, and verified using foundry processes.

The voltage input to the MN3 transistor in the DPI circuit, which includes the memristor’s weight value, generates the total current, *I*_tot_. This current is the result of the discharge of *C*_syn_, with the amount of discharge varying in accordance with the magnitude of the signal at the gate of MN3. A change in the voltage applied to the MP2 transistor, *V*_syn_, will result in a corresponding alteration of the gate voltage, which in turn will affect the current flowing through the synapse, *I*_syn_. The decay time (τ) of *I*_syn_ and the charging time of *V*_syn_ can be modified by adjusting the *V*_syn,tau_ value at the gate of the MP1 transistor, thereby controlling *I*_tau_. A portion of the generated *I*_syn_ flows to the ground through MN5, while the remainder charges *C*_mem_. The voltage across *C*_mem_ (*V*_membrane_) represents the post-neuron’s membrane potential, and the membrane τ can be adjusted by controlling the gate voltage *V*_mem,tau_ of MN5.

Consequently, when a spike signal occurs in the 1T1R structure, a voltage signal incorporating the memristor’s weight value is generated through the utilization of the TIA. This signal is then converted into the *I*_syn_ current via the DPI circuit, which discharges *C*_mem_, thereby altering the *V*_membrane_ signal. From a mathematical perspective, the synapse exhibits both a charge time constant and a discharge time constant. The membrane potential rises in accordance with *I*_syn_, excluding the influence of the discharge current *I*_D0_ caused by the MN5 transistor.

The operation of the circuit can be mathematically described by Equations 6, 7, which account for the charge and discharge of the synapse. Ultimately, the change in membrane potential is expressed by Equation 8.


(6) $$I_{syn}\left(t\right)=\frac{I_{\mathrm{gain}}I_{\mathrm{tot}}}{I_{\tau }}\left(\;1-e^{-\frac{\left(t-t_{i}\right)}{\tau_{syn}}}\right)+I_{syn}e^{-\frac{\left(t-t_{i}\right)}{\tau_{syn}}}$$

(7) $$I_{syn}\left(t\right)=I_{syn}e^{-\frac{\left(t-t_{i}\right)}{\tau_{syn}}}$$

(8) $$I_{\mathrm{gain}}=I_{0}e^{-\frac{k\left(V_{thr}-V_{dd}\right)}{u_{T}}}\mathrm{at}\;PMOS^{’}s\;\mathrm{subthreshold}$$

(9) $$\mathrm{Membrane}:\frac{dV_{C}}{dt}=\frac{I_{syn}}{C}-\frac{I_{D0}}{C}\;\exp\left(\frac{V_{C}-V_{G}-|V_{th}|}{ku_{T}}\right)$$

In practice, factors such as the subthreshold slope factor (*k*) and the thermal voltage (*u*_T_) were employed due to the use of actual transistors (Streetman and Banerjee, 2000). The aforementioned set of equations indicates that the dynamic rise and fall behaviors of the synapse current, as described by Equations 6, 7, and the membrane potential Equation 9 in the form of the LIF neuron, can provide a similar operational output to that simulated in PyTorch, provided that the appropriate parameters synapse current tau (*τ*_syn_), membrane potential tau (according to *I*_D0_), *V*_th_, *C*_mem_ are selected.

### 3.4 Implementation of memristive spiking neural networks for inference

**Figure 7:**
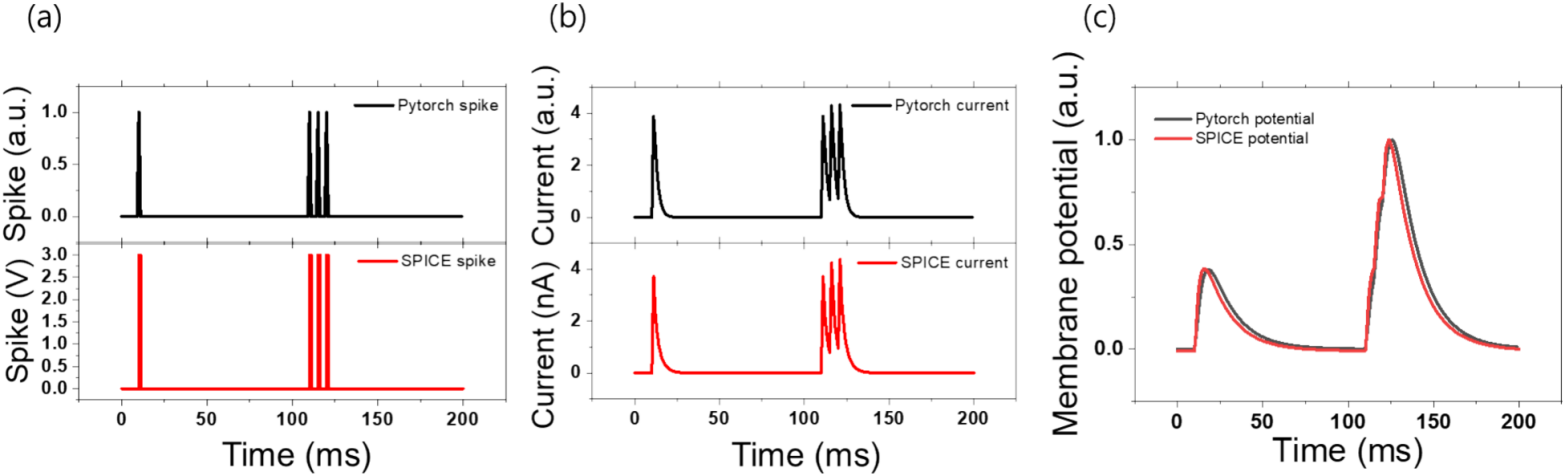
The following results were obtained from both PyTorch and SPICE simulations, showing the voltage spike signal, synaptic current, and membrane potential. Panel **(A)** illustrates the shape of the voltage spikes that occur at 10, 110, 115, and 120 ms in both PyTorch and SPICE simulations. Panel **(B)** shows the changes in synaptic current in response to the spike events in the PyTorch simulation, where 𝛕_rise_ was set to 0.5 ms and 𝛕_fall_ was set to 2.0 ms. Panel **(C)** illustrates the resulting membrane potential graph, with 𝛕_membrane_ set to 15 ms (converted to arbitrary units for relative comparison).

As stated above, the dynamic spiking neural network behavior can be simulated in PyTorch using the set of the equations (Equations 1–5) and emulated in SPICE using the hardware shown in Figure 6, respectively. With the proper choices of the parameters of the circuit, the spiking neural network behavior of the circuit can be matched to that of PyTorch simulation. Input spike signals fired at 10, 110, 150, and 200 ms as shown in Figure 7A were used both of the PyTorch and SPICE simulations. In PyTorch, the time step is defined in 1 ms increments, resulting in an impulse-like firing structure. In SPICE, the signal was generated as a pulse with a rise time of 100 μs, a fall time of 100 μs, and a pulse width of 900 μs. When these pulses were applied, the changes in *I*_syn_ were observed as shown in Figure 7B. In PyTorch, τ_rise_ was set to 0.5 ms and τ_fall_ was set to 2.0 ms. To mimic this in SPICE, *V*_syn,tau_ was set to 1.44 V and *C*_syn_ was set to 260 pF. Lastly, Figure 7C is provided to verify the accuracy of the following pattern, the membrane potential in PyTorch used *τ*_mem_ of 15 ms, while in SPICE, *V*_mem,tau_ was set to 0.1 V and *C*_mem_ was set to 260 nF. The result showed a time error of approximately 2.1% relative to the maximum potential in the membrane potential, achieving a satisfactory match.

**Figure 8:**
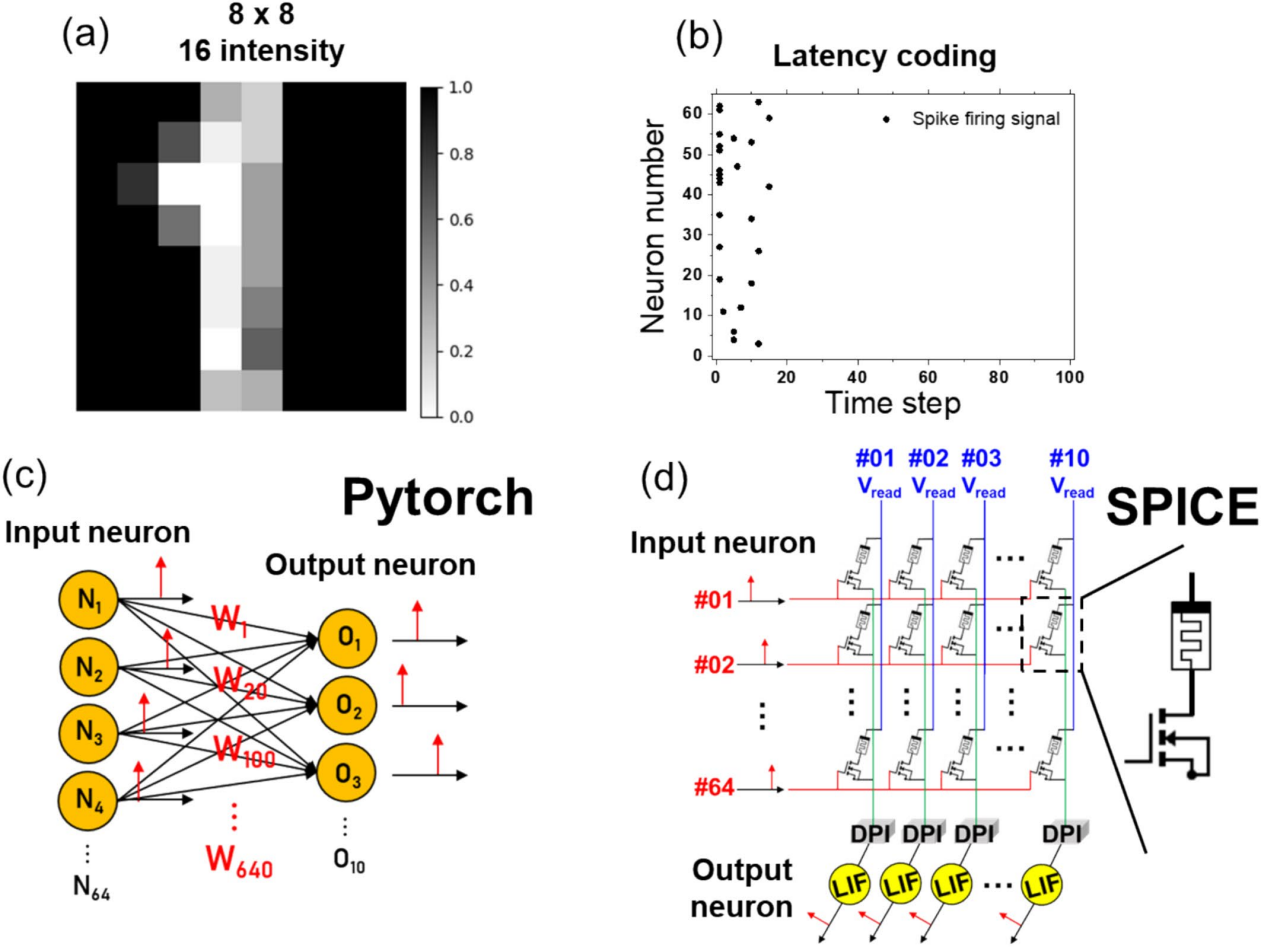
**(A)** The input data is a reduced 8×8 MNIST image with 16 intensity levels representing digit patterns. **(B)** Based on the intensity of the input image, the spike firing time is calculated using the Equation 10, and the neurons that fire at each time step are plotted. **(C)** To verify the accuracy of MNIST pattern recognition in PyTorch, the signals are converted using latency coding. These signals are fed into an input neuron layer with 64 inputs, 640 weights, and 10 output neurons. The accuracy is then assessed based on the membrane potential of the output neurons. **(D)** In SPICE, the spike signals from 64 input neurons are applied to a 1T1R gate. The current, modulated by the read voltage and the memristor conductance, is converted to synapse current through a DPI circuit, and the final output neuron’s membrane potential is used to evaluate the accuracy.

The prepared components were employed in the performance of pattern recognition, which is an exemplar of edge computing. An 8 × 8 handwritten digit image with 16 intensity levels, as illustrated in Figure 8A, was employed, and the values were transformed through latency coding.


(10) $$\mathrm{Latency}\;\mathrm{coding}:t_{\text{max}}\cdot \ln\left(\frac{x}{x-x_{thr}}\right)$$

The equation for latency coding is presented in Equation 10. Latency coding employs *t*_max_ as the maximum value, with the firing time determined when the pixel intensity, *x*, surpasses the threshold (*x*_*thr*_). Upon applying a *t*_max_ of 20 and a thr of 0.3 to the image in Figure 8A, the neurons firing at each time step are determined, as illustrated in Figure 8B. Subsequently, the latency-coded spike signals were introduced as input into the PyTorch neurons, as illustrated in Figure 8C. Subsequently, the signals were conveyed through a 64 × 10 configuration of weights to the output neurons. By examining the membrane potential signals within the output neurons, we were able to verify whether the neuron corresponding to the correct label exhibited the highest membrane potential. Upon completion of the PyTorch simulation, the 64 × 10 weights were extracted and input into the normalized state variable parameters (ranging from 0 to 1) of the SPICE memristor model. Upon inputting the latency-coded spikes as gate voltages to the 1T1R devices, a net current was generated by *V*_read_, set at 50 mV. The current was then converted into a voltage signal by the TIA and subsequently input as the gate voltage to the DPI circuit. Based on the input signal, *I*_syn_ was generated, and ultimately, the maximum value of the membrane potential of the LIF neuron over a 100 ms time period was employed as the criterion for verifying accuracy. The SPICE implementation structure is depicted in Figure 8D.

**Figure 9:**
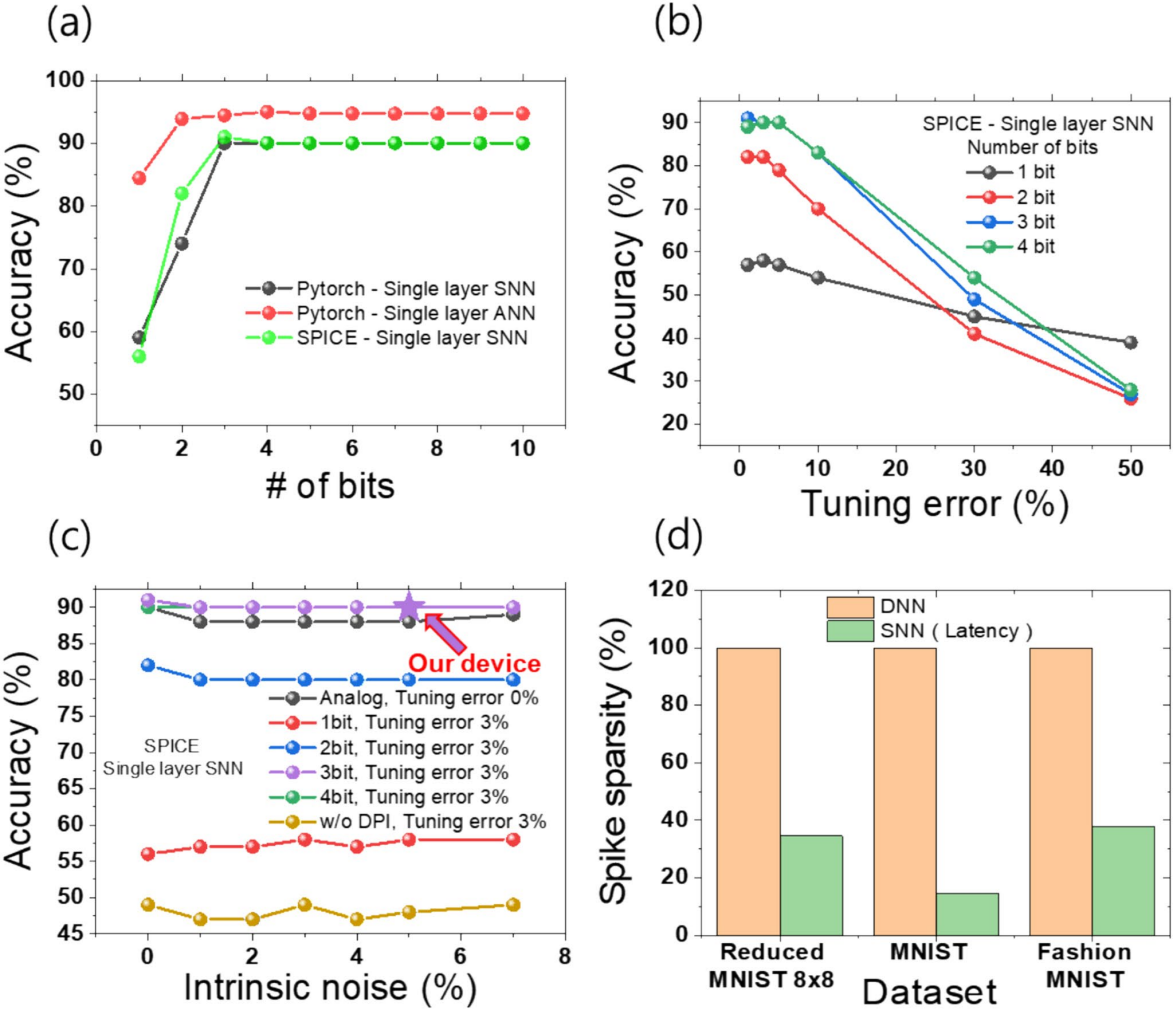
We summarize the accuracy and sparsity of pattern recognition based on the PyTorch network and SPICE circuit simulations. **(A)** The accuracy was evaluated as a function of the synapse bits. **(B)** A graph was generated by performing SNN simulations in SPICE, accounting for tuning error (i.e., mapping accuracy) and synapse bits. **(C)** PyTorch and SPICE simulations were conducted, considering the combined effects of intrinsic noise, a 3% tuning error, and varying synapse bits. Our experimental results from measured device data, with 3-bit precision, 3% tuning error, and 5% intrinsic noise, achieved 90% accuracy. **(D)** A comparison table between latency-coded SNN and DNN highlights the spike firing sparsity. While variations exist depending on the dataset, all three representative examples show spike sparsity within 40%.

As illustrated in Figure 9A, the simulation outcomes demonstrate the precision outcomes as a function of the number of bits. The results demonstrate that for both PyTorch ANN and PyTorch SNN, as well as SPICE SNN, the accuracy reaches a saturation point at 3 bits or more. Notably, both PyTorch SNN and SPICE SNN exhibit a saturation accuracy of 90%. This finding is consistent with other literature on bit dependence, indicating that a certain number of bits beyond a threshold are necessary, but not unlimited. The discrepancy in accuracy between PyTorch SNN and SPICE SNN at 2 bits and 3 bits can be attributed to the differing sizes of the training datasets. The PyTorch SNN utilized 1,124 images from the training data set, whereas SPICE simulations were conducted on only 100 images due to time constraints, resulting in a sampling bias. In light of the fact that the conductance results obtained from the 1T1R devices yielded nine distinct states, it may be reasonably assumed that accuracy should not differ significantly with more than three bits of conductance states. Figure 9B illustrates that, although not observed in PyTorch SNN, real hardware implementation demonstrated a range of tuning errors due to the inability to achieve the target conductance with precision. The aforementioned tuning error affects the accuracy of the system, with a tolerance of up to 5% exhibiting no significant decline in accuracy. However, beyond a 10% tuning error, there is a notable reduction in the accuracy of the system. Although the absolute accuracy is lower for a one-bit binary representation, it demonstrates a higher tolerance to tuning errors. Figure 9C examines the influence of intrinsic noise on accuracy without constraints on bit precision or tuning error, with a 3% tuning error from our device. In the SPICE model, noise was defined as a time-dependent function, with values ranging from 0 to 7% relative to a 0% noise baseline. Although intrinsic noise has a slight impact on accuracy, the overall system demonstrates tolerance, as the cumulative effect of the DPI circuit and its function as a low-pass filter effectively suppresses the noise (Bartolozzi et al., 2006).

These results are in line with the study of chemical synapses, which show that despite the presence of intrinsic noise, chemical synapses which our DPI circuit mimics enhance the system’s coherence through the selective reduction of unnecessary correlations, thereby suggesting more robust and reliable information processing compared to electrical synapses (Balenzuela and García-Ojalvo, 2005). The LIF neuron exhibits low-pass filter behavior due to the cumulative effects of the RC circuit. However, parameter tuning can suppress the neuron’s operation. To address this, we implemented a tunable and stable low-pass filter for noise attenuation using a differential pair integrator (DPI) circuit. Additional data, presented in Supplementary Figure S7 and Supplementary Table S3, demonstrates the impact of intrinsic noise on the membrane potential. Despite a noise level of 7%, the peak difference in membrane potential is approximately 3%. Consequently, as shown in Figures 2D, 6, the proposed Cu device exhibits intrinsic noise within 5% and a tuning error around 3%, allowing for the implementation of analog behavior with a precision exceeding 3 bits. Additional detailed results for bit count and tolerance are presented in Figure 9C. As a result, a final inference accuracy of 90% can be achieved. In terms of intrinsic noise and tuning error, the array using only memristors demonstrated greater stability compared to traditional configurations (Park et al., 2022). In previous DNN-based research, significant accuracy degradation was observed due to system tuning errors and the intrinsic noise of the devices (such as RTN noise). Under conditions similar to ours, with 5% intrinsic noise and 3% tuning error, systems with conductance below 80 μS showed an accuracy decrease of over 20% (Park et al., 2022). However, in our research, we utilized a high-performance memristor that suppresses intrinsic noise to within 2% even in the conductance range below 100 μS. Even when assuming 5% noise in simulations, we constructed a noise-tolerant inference system by leveraging the cumulative effects of the DPI circuit to attenuate noise. Additionally, we employed a 1T1R configuration to suppress sneak path currents, thereby preventing the overlap of errors and noise during actual operation (Youssef et al., 2021).

This study builds upon the design of SNN inference accelerator for power efficiency, extending it to the implementation of SNN edge computing functionalities. This is demonstrated through the reduction of MNIST 8 × 8 simulations. In a SNN-based edge computing system employing latency coding, spikes from low-intensity pixels below the threshold are not processed. The spike sparsity of latency coding is demonstrated in Figure 9D. Spike sparsity refers to the average number of neurons that fire per image. When the operation of all neurons in a DNN is considered 100%, the SNN, due to latency coding, shows a spike sparsity of less than 40% across datasets such as Reduced MNIST, MNIST, and Fashion MNIST, although the exact sparsity varies depending on the dataset. Furthermore, the analysis process and results were reflected in Supplementary Figure S8 through power analysis of the SPICE circuit. Although the circuit does not exhibit the highest power efficiency, we compared its power efficiency with that of research from other groups outside the state-of-the-art level.

Although the actual simulation was conducted using a single-layer neural network, the power consumption was calculated based on a more complex multilayer structure. The network for the Reduced MNIST (8 × 8) dataset comprised 64 input neurons, 21 hidden neurons, and 10 output neurons. The network was composed of 28 × 28 input neurons, 256 hidden neurons, and 10 output neurons for the MNIST and Fashion MNIST datasets. In memristive neural networks, power consumption is primarily attributed to the access of memristors by spikes. With sparsity levels within 40% in the input layer and within 6% in the hidden layer, the efficiency increases as the number of layers grows. Previous studies have also reported a reduction in spikes in multilayer structures (Chowdhury et al., 2022; Dampfhoffer et al., 2022).

Furthermore, SNNs benefit from sparse input signals, which reduces the current burden on the driving circuit and enables temporal operation. This allows for intermittent inference and lower idle power consumption due to event-driven operation. From a power and circuit perspective, memristor-based deep neural network research has revealed considerable power consumption and noise susceptibility in analog-to-digital converter (ADC) and digital-to-analog converter (DAC) components (Moro et al., 2022). In contrast, the use of DPI and LIF neurons eliminates the necessity for an ADC and a DAC, thereby offering a distinct advantage (Li et al., 2023).

## 4 Conclusion

In order to overcome the limitations of power consumption that are inherent to the traditional von Neumann architecture, which is characterized by a bottleneck, ASIC systems have been proposed. Among these, there is a particular need for research on the hardware accelerator in order to address the issue of bottlenecks. We put forth the proposition of SNN edge computing, wherein memristors are employed in a PIM capacity. We provide a detailed account of the operational and utilitarian aspects of the requisite components, including memristors, transistors, TIAs, and LIF neurons.

In particular, we elucidated the distinctions between MOS and MOD configurations when integrating memristors and transistors into a 1T1R structure, emphasizing the challenges associated with conventional resistors and their categorization according to their set and reset behavior when employed as memristors. By delineating the selection criteria for suitable transistors for our memristors, we enhanced the comprehension of 1T1R configurations and furnished practical directives for implementation.

Following a comprehensive examination of the attributes of individual devices, we devised a SPICE hardware simulation to emulate PyTorch simulations, thereby demonstrating that devices exhibiting conductance levels of 3 bits or more do not exhibit notable discrepancies in accuracy. Moreover, we addressed the implementation challenges posed by tuning errors, demonstrating that a tolerance within 5% enhances feasibility. The cumulative effect and low-pass filter functionality of the DPI circuit mitigated the intrinsic noise, allowing for up to 7% noise without significantly affecting accuracy.

In addition to the superior hardware design, the benefits of SNNs, such as latency coding and reduced load due to temporal operation, were also leveraged. The elimination of the necessity for ADC and DAC resulted in a notable reduction in power consumption and enhanced resilience to noise. While PIM has not yet supplanted traditional computing, ongoing research into high-quality hardware and software technologies is anticipated to facilitate the deployment of memristor-based SNN analog computing.

## Data Availability

The raw data supporting the conclusions of this article will be made available by the authors, without undue reservation.
