# Supplementary material for: Design of CMOS-memristor hybrid synapse and its application for noise-tolerant memristive spiking neural network
Source: Front Neurosci. 2025 Mar 5;19:1516971. doi: 10.3389/fnins.2025.1516971 (PMC11920157; doi:10.3389/fnins.2025.1516971)
Supplement: Supplementary file 1 [file Data_Sheet_1.docx]

Supplementary Information

Design of CMOS-memristor hybrid synapse and its application for noise-tolerant memristive spiking neural network

Jae Gwang Lim^1,2^, Sang Min Lee^1,3^, Sung-jae Park^1,3^, Joon Young Kwak^4^, Yeonjoo Jeong^1^, Jaewook Kim^1^, Suyoun Lee^1^, Jongkil Park^1^, Gyu Weon Hwang^1^, Kyeong-Seok Lee^1^, Seongsik Park^1^, Byeong-Kwon Ju^2,3^, Hyun Jae Jang^1,*^, Jong Keuk Park^1,*^, Inho Kim^1,*^

*^1^Center for Neuromorphic Engineering, Korea Institute of Science and Technology, Seoul 02792, South Korea*

*^2^School of Electrical Engineering, Korea University, Seoul 02841, South Korea*

*^3^Department of Micro/Nano Systems, Korea University, Seoul 02841, South Korea*

*^4^Division of Electronic and Semiconductor Engineering, Ewha Woman’s University, Seoul 03760, South Korea*


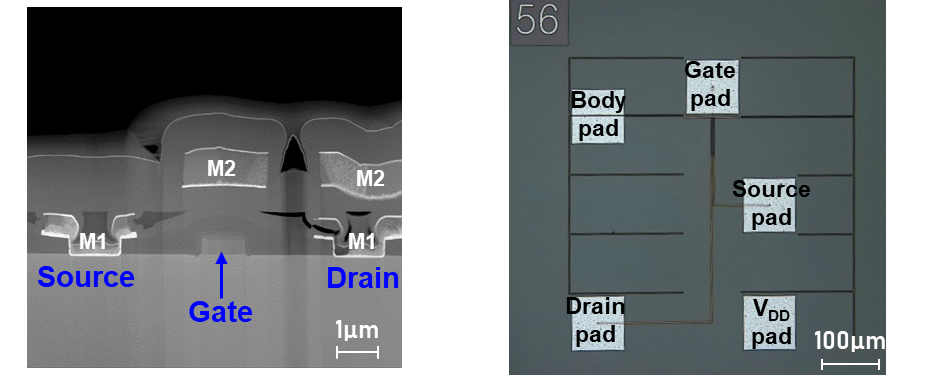


**Supplementary Figure S1.** Left: TEM image of the transistor. Right: OM image of the transistor.

The dynamic memdiode model reported by Fernando Leonel Aguirre in 2022[1, 2] was employed. The model comprises two principal equations: one governing the I-V characteristics and the other governing the state variable of the memristor. The primary and subsidiary equations are presented in detail below.

$I\left( V_{c} \right)=I_{0}\sinh\left[ \alpha\left( V_{c}-R_{s}I \right) \right]$ (1)

$\alpha_{0}=\left( \alpha_{on}-\alpha_{off} \right)\lambda+ \alpha_{off}$ (2)

$I_{0}=\left( I_{on}-I_{off} \right)\lambda+ I_{off}$ (3)

$R_{s}=\left( R_{off}-R_{on} \right)\lambda+ R_{on}$ (4)

$\frac{d\lambda}{dt}=\frac{1-\lambda}{\tau_{s}(\lambda,V_{c})}- \frac{\lambda}{\tau_{r}(\lambda, V_{c})}$ (5)

$\tau_{s}=\exp( -\eta_{s}(V_{c}-V_{s}(\lambda)), \tau_{r}=\exp( \eta_{r}\lambda^{\gamma}(V_{c}-V_{r}))$ (6)

In accordance with the tenets of equation (1), the current as a function of V_c_ is determined. This process entails the sub-parameters and the preceding current state, effectively establishing the prospective trajectory of change and thereby reflecting the current memory. The sub-parameters include α_0_, I_0_, and R_s_, which correspond to the nonlinear characteristic, the current window, and the memristor resistance, respectively. The aforementioned sub-parameters serve to determine the current operating conditions, based on the state variable λ. The state variable is determined using equation (5), with the values of τ_s_ and τ_r_ calculated in accordance with equation (6). In equation (6), the set voltage, V_s_(λ), is varied in accordance with the state variable λ, thereby facilitating the implementation of snapback. The influence of λ on V_s_ can be disregarded if it is ignored. The magnitude of the snap-forward phenomenon can be modified by the γ value. When γ is set to zero, the snap-forward phenomenon can be disregarded. The compact model was constructed based on the observed behavior of the memristor, and the IV curve in supplementary information S2. As seen in the IV curve, the reset behavior above 1V is observed to be abrupt, which differs from actual measurements. In this model, we used the current and resistance to fit the complex memristor characteristics. The on/off ratio for current is 5000, and the resistance ratio is 290. Since the compact model is fundamentally based on resistance, it operates with resistance as the primary parameter in accordance with Ohm’s law. While it cannot be said that only the current variable operates during the set process and only the resistance variable operates during the reset process, current dominates as the more significant parameter during the set operation, limiting the current in an analog manner. On the other hand, during the reset process, although the resistance dominates in the HRS state to simulate the behavior, the smaller ratio compared to the current makes it difficult to fully replicate the analog behavior. The parameters of the compact model used in the study are summarized in Supplementary Table S1. Currently, the system has been used only for inference, so the impact on accuracy is minimal. However, issues may arise when performing the reset write operation. Therefore, parameter tuning or the application of a model capable of implementing analog behavior for set/reset operations using a single parameter may be worth exploring.

**
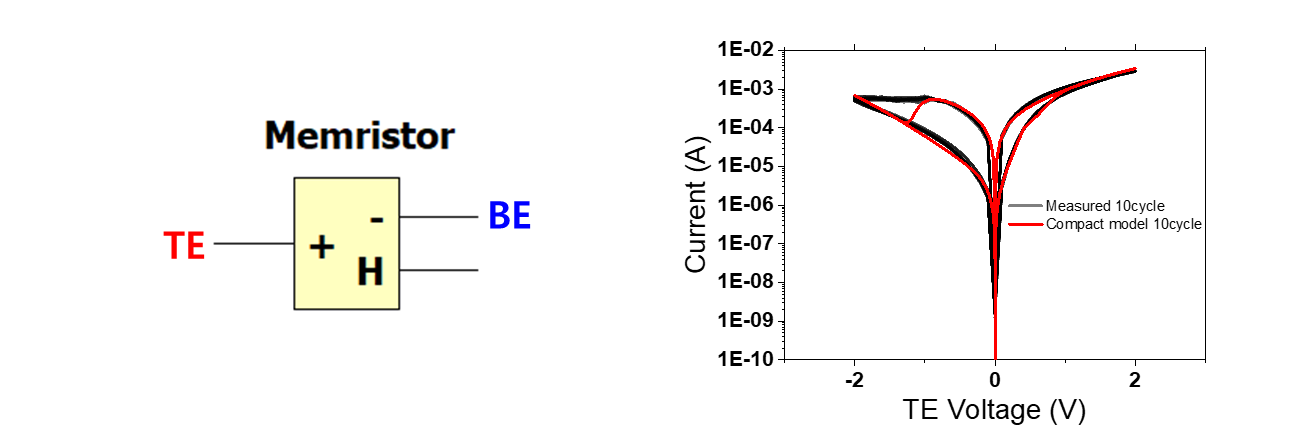
**

**Supplementary Figure S2.** Left: Memristor SPICE symbol. Right: IV measurement data from the actual device and the SPICE model.

**Supplementary Table S1.** Summary of the parameters for the memristor compact model.

| **Parameters** | **Value** | **Parameters** | **Value** |
| --- | --- | --- | --- |
| *I_on_* | 5 mA | *Initial* λ (λ_0_) | 0 |
| *I_off_* | 1 μA | *V_s_* | 0.3 V |
| *R_on_* | 100 Ω | *V_r_* | -0.2 V |
| *R_off_* | 29 kΩ | η_s_ | 40 |
| *a_on_* | 5,000 | η_r_ | 40 |
| *a_off_* | 100,000 | *γ* | 1000 |

According to the description in Supplementary Figure S2, the model enables the implementation of complex memristive behavior through the adjustment of the I_0_ parameter and R_s_. As shown in Supplementary Figure S1, the parameters were finely tuned to fit the memristor's behavior. However, when both factors were adjusted simultaneously in terms of variation, the behavior exhibited amplified deviations. Therefore, the current was controlled through the I_off_ and I_on_ parameters, while the threshold voltage was varied to mimic the cycle-to-cycle and device-to-device variations observed in actual devices.


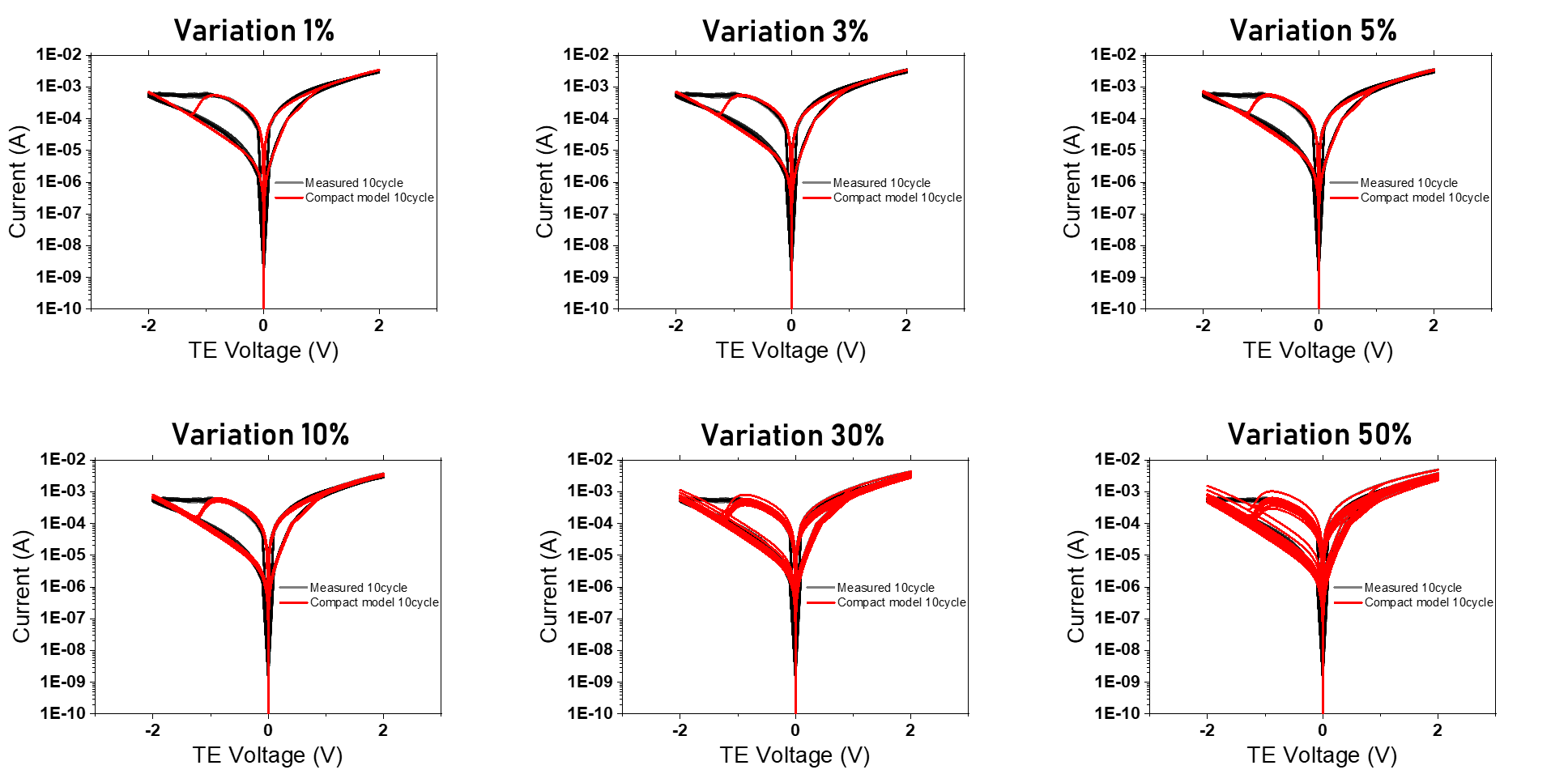


**Supplementary Figure S3.** Results of a 10-cycle IV sweep performed while varying the SPICE compact model.

The transistor's gate voltage and W/L ratio significantly influence its on-resistance and I-V characteristics, which, in turn, affect the behavior of the 1T1R structure, especially with respect to the memristor's resistance. These factors lead to variations in the voltage applied to the memristor (V_memristor_). To evaluate these effects, we used a transistor compact model with a fixed resistance attached and analyzed how the voltage applied to the memristor changes under 1T1R conditions. The V_DD_ pad was set to 3V, while the gate voltage varied from 0.8V to 3V, and the transistor's W/L ratio was adjusted across six configurations. The results are shown in Figure S4, with the differences in configurations illustrated in Figures S4(a)-(c) for one case and Figures S4(d)-(f) for the other. Simulations were conducted across three resistance levels for the memristor 1kΩ, 10kΩ, and 100kΩ. To satisfy the primary requirement of applying a 2V set voltage, we selected the MOD set configuration shown in Figure 3(b). Furthermore, we utilized a transistor with a W/L ratio of 65 to facilitate voltage application to the memristor even at lower gate voltages.


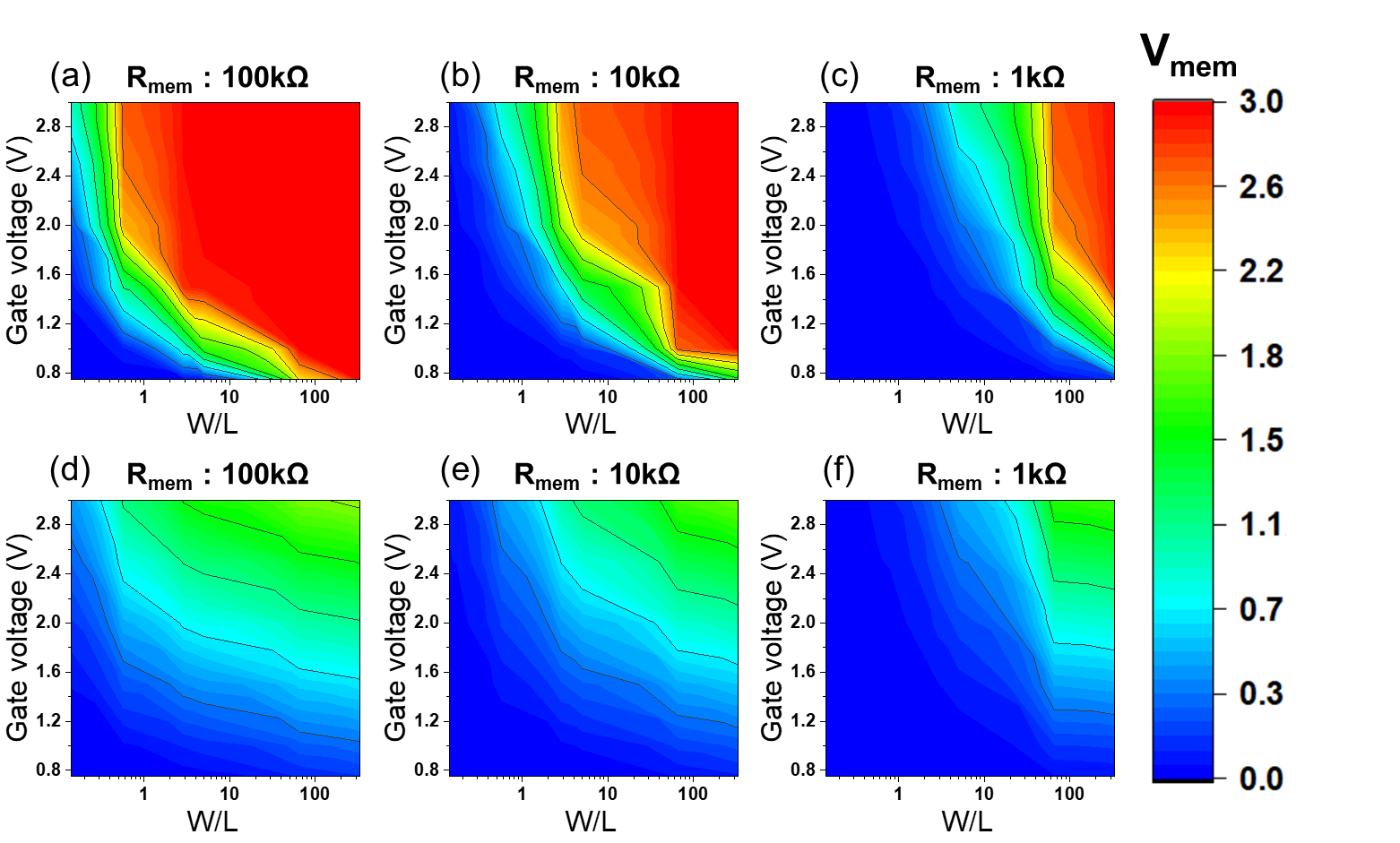


**Supplementary Figuer S4.** (a), (b), and (c) present contour graphs of the voltage across the memristor as a function of varying W/L ratios and gate voltages for six types of transistors in the MOD set and MOS reset scenarios. In these simulations, the memristor resistance is set to 100kΩ in (a), 10kΩ in (b), and 1kΩ in (c) for the 1T1R configuration. Similarly, Figures (d), (e), and (f) show contour graphs of the voltage across the memristor under the MOD reset and MOS set scenarios for the same six transistor configurations. The memristor resistance is set to 100kΩ in (d), 10kΩ in (e), and 1kΩ in (f), also in the 1T1R simulation.

**
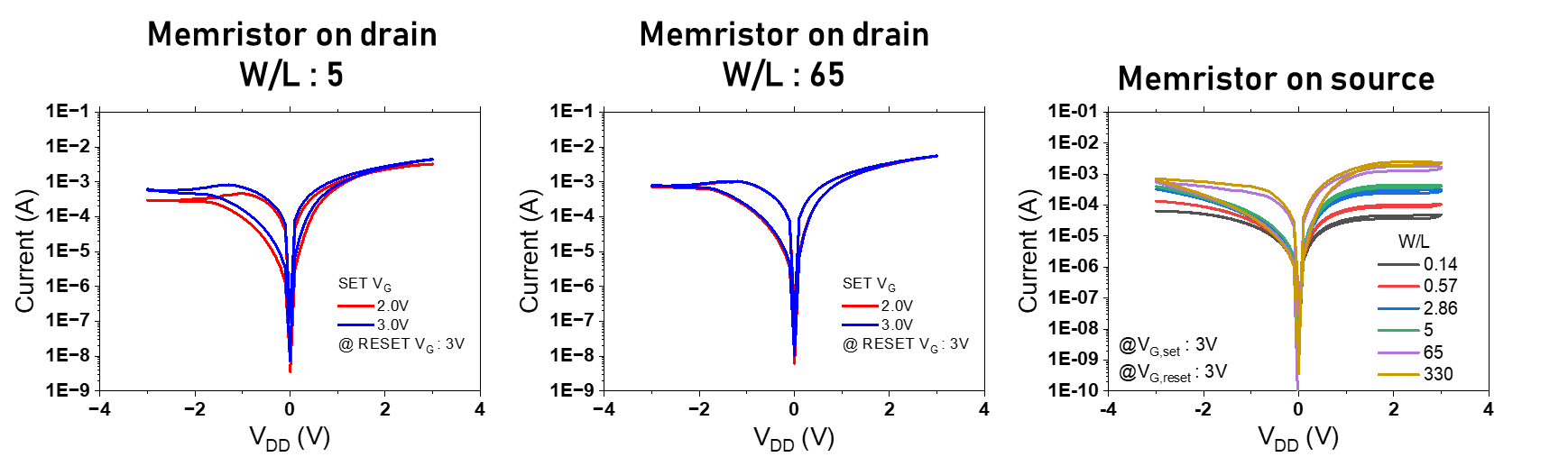
**

**Supplementary Figure S5.** IV curve graphs as a function of W/L for both MOS and MOD structures.

**Supplementary Table S2.** Summary table of on/off ratio variations as a function of W/L.


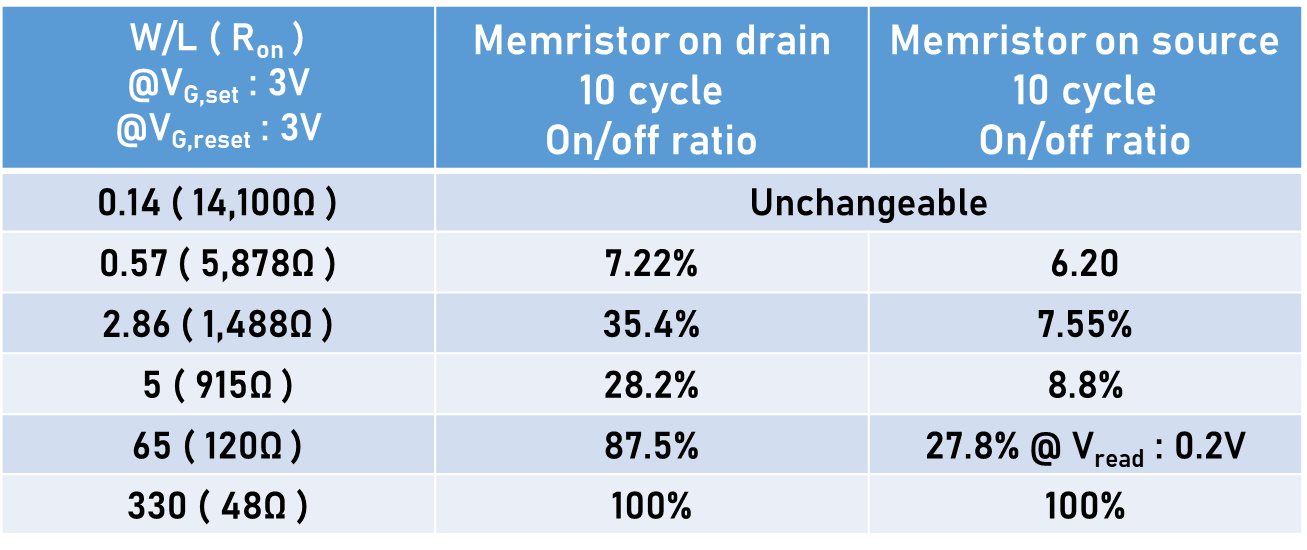


**
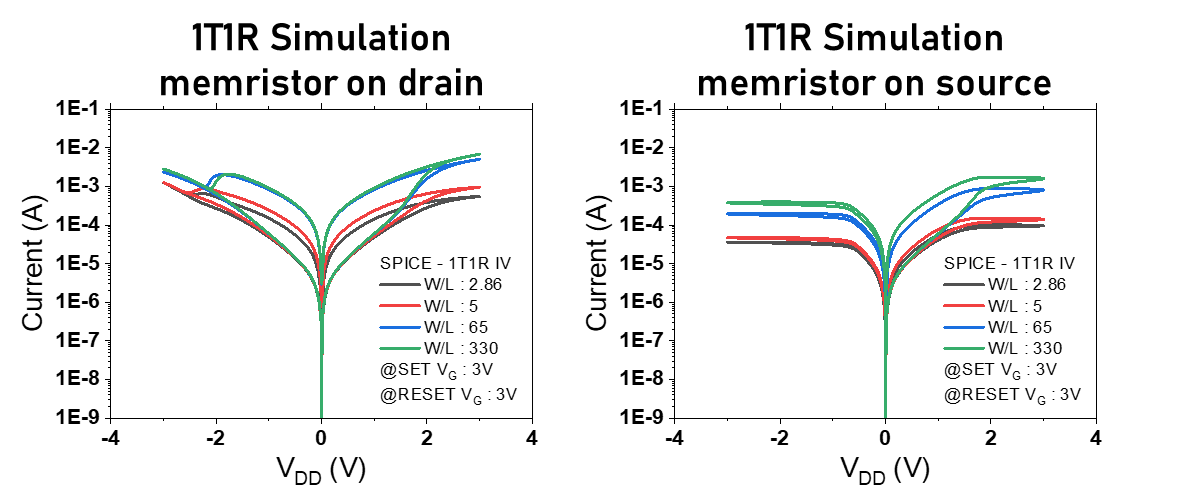
**

**Supplementary Figure S6.** IV curves from 1T1R simulations of MOS and MOD structures using a compact model, with W/L variations applied.

**Supplementary Figure S7.** Membrane potential under RTN noise conditions from SPICE inference accelerator simulations, conducted with a 3% tuning error and 3-bit precision.

**Supplementary Table S3.** A table based on the data from Supplementary Figure S6 showing that all four peaks have a noise level of only 3%


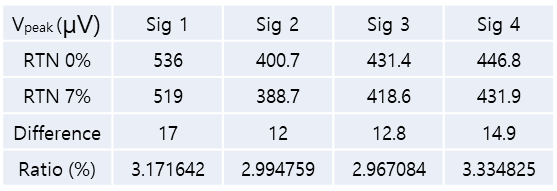


Although we intended to consider power consumption during learning, our system primarily operates as an accelerator, focusing on mapping followed by inference. Therefore, we compared the power consumption of inference in systems using memristors and Spiking Neural Networks (SNNs). To calculate the power consumption, we examined the power supply of the SPICE circuit elements, which are mostly powered by voltage sources, and computed the power consumption of these voltage sources. The result showed that the data within 5 ms of performing inference for a single handwritten digit had the most dominant impact on the final result. Consequently, we calculated the power consumption related to the spikes generated when a latency-coded handwritten digit image is input. Although power consumption varied depending on the specific handwritten digit image, we chose an image with average power consumption to represent the calculation.

The analysis was divided into three main areas: the 1T1R array, the section where current is converted to voltage and amplified, and the DPI and LIF circuits. The resulting power consumption is described in terms of W/operation, where one inference operation is defined as requiring 5 ms, corresponding to the total time for final verification of the operation. During a single inference operation, the power consumption in the memristor array is measured as 20 μJ, while the DPI and LIF circuits consume 10 μJ. Additionally, the amplifier circuit consumes 2.5 mJ, with leakage in the amplifier circuit accounting for the largest portion (98.8%) of the total power consumption, making it the most significant contributor to overall power usage.

The current system is not aimed at proposing the highest-performing system but serves as a research direction and a design guideline. Due to practical limitations, the results are not optimal. The primary cause of this is that if a memristor with a higher resistance state were used specifically one with 1,000 times the resistance of the memristor array power consumption could be reduced to around 20 nJ. Furthermore, the current 1 ms pulse width is relatively long, as the focus was on mimicking PyTorch learning in hardware, without using shorter pulse widths. Memristor devices have the potential to operate at sub-nanosecond levels, so improvements in these devices could lead to a reduction in power consumption. If these two conditions are applied, we expect to achieve results on the order of 20 pJ/operation (atto: 10^-18^) (operation = 5ns).

From a circuit perspective, the system is currently designed based on 150 nm TSMC transistors, with the 1T1R array using 500 nm ETRI foundry transistors. These transistor characteristics are not state-of-the-art, so using more advanced process technologies or actual transistors could significantly reduce power consumption.

Finally, in the amplifier section, the use of commercial operational amplifiers (op-amps) in hardware implementation of a neuromorphic system resulted in high leakage currents. Without considering the op-amp leakage, the power consumption was calculated to be 30 μJ. Including the op-amp leakage, the power consumption increased to 2.53 mJ. Although this is lower than the state-of-the-art level of ~20 pJ for SRAM-based systems such as Loihi[3], It remains lower than the reported power consumption per inference of 4 mJ and 278 mJ in small-scale SRAM-based systems fabricated using 180 nm and 65 nm technologies, respectively [4][5]. Additionally, in RRAM-based research, the power consumption in SNN-based 5x4 image classification was reported to be 2.9 mJ[6], which is higher than our results.

Our system’s power efficiency is currently limited by the significant power loss due to the leakage of commercial op-amps. The simplest solution to improve this is to apply low-power op-amps. In advanced research, designing custom CMOS-based amplifiers could be another potential solution. Despite the current limitations caused by op-amp leakage accounting for 98.8% of power consumption, we anticipate significant improvements in the overall neuromorphic hardware system through advancements in circuits, memristor devices, pulse width modulation, and process technology. With these improvements, a more advanced system could be realized when the neuromorphic hardware is physically implemented.

**Supplementary Figure S8.** A pie chart displaying the overall power consumption values and their corresponding proportions.

**References**

[1] F. L. Aguirre, J. Suñé, and E. Miranda, "SPICE implementation of the dynamic memdiode model for bipolar resistive switching devices," Micromachines, vol. 13, no. 2, p. 330, 2022.

[2] P. A. Lázaro, I. J. Gallo, J. R. Aranda, A. d. B. García, G. B. Juan, and F. J. Molinos, "Design and simulation of memristor-based neural networks," arXiv preprint arXiv:2306.11678, 2023.

[3] Davies, Mike, et al. "Loihi: A neuromorphic manycore processor with on-chip learning." *Ieee Micro* 38.1 (2018): 82-99.

[4] G. Indiveri, F. Corradi, and N. Qiao, “Neuromorphic architectures for spiking deep neural networks,” in 2015 IEEE International Electron Devices Meeting (IEDM). IEEE, 2015, pp. 4–2.

[5] Y.-H. Chen, T. Krishna, J. S. Emer, and V. Sze, “Eyeriss: An energyefficient reconfigurable accelerator for deep convolutional neural networks,” IEEE journal of solid-state circuits, vol. 52, no. 1, pp. 127–138, 2016

[6] Nowshin, Fabiha, and Yang Yi. "Memristor-based deep spiking neural network with a computing-in-memory architecture." *2022 23rd International Symposium on Quality Electronic Design (ISQED)*. IEEE, 2022.
